# Chloride-Anion-Templated Synthesis of a Strapped-Porphyrin-Containing Catenane Host System

**DOI:** 10.1002/chem.201502721

**Published:** 2015-10-28

**Authors:** Asha Brown, Matthew J Langton, Nathan L Kilah, Amber L Thompson, Paul D Beer

**Affiliations:** a Department of Chemistry, University of Oxford, Chemistry Research Laboratory Mansfield Road, Oxford, OX1 3TA (UK); b School of Physical Sciences – Chemistry, University of Tasmania Hobart, Tasmania, 7001 (Australia)

**Keywords:** anion binding, catenanes, porphyrins, self-assembly, supramolecular chemistry

## Abstract

The synthesis, structure and anion-recognition properties of a new strapped-porphyrin-containing [2]catenane anion host system are described. The assembly of the catenane is directed by discrete chloride anion templation acting in synergy with secondary aromatic donor–acceptor and coordinative pyridine–zinc interactions. The [2]catenane incorporates a three-dimensional, hydrogen-bond-donating anion-binding pocket; solid-state structural analysis of the catenane⋅chloride complex reveals that the chloride anion is encapsulated within the catenane’s interlocked binding cavity through six convergent CH⋅⋅⋅⋅Cl and NH⋅⋅⋅Cl hydrogen-bonding interactions and solution-phase ^1^H NMR titration experiments demonstrate that this complementary hydrogen-bonding arrangement facilitates the selective recognition of chloride over larger halide anions in DMSO solution.

## Introduction

[*n*]Catenanes, a class of mechanically bonded molecules comprising *n* interlocked ring components,^1^ have received ever-growing attention over recent decades on account of their interesting topologies and aesthetic appeal, in addition to their potential applications as molecular machines,^2^ imaging agents,^3^ host systems^4^ and functional nanomaterials.^5^ However, despite the widespread interest in catenane compounds, their synthesis remains challenging, usually relying on the use of interweaving templating interactions to organise the molecular precursor components in an orthogonal manner, before performing the final ring-closing step. Since Sauvage’s pioneering use of a Cu^I^-directed orthogonal assembly strategy,^6^ much synthetic effort has been devoted to the development of new and efficient template-directed protocols for the preparation of catenanes. Although coordinate metal–ligand bonds remain the most widely exploited templating interactions,^7^ a variety of alternative noncovalent interactions, including π–π interactions,^8^ hydrogen bonding,b8c, 9 radical–radical interactions,^10^ halogen bonding,^11^ and solvatophobic effects,^12^ have been successfully applied to catenane synthesis.

Our group^13^ and others^14^ have demonstrated that anions can also be effectively employed as discrete interweaving templates during catenane synthesis, and that the preorganised three-dimensional binding pockets contained within the resultant interlocked architectures can subsequently be exploited for selective anion-recognition purposes.^15^ Incorporation of a suitable optical or redox-active reporter group can give rise to systems that produce a signalling response upon complexation of the target guest species. However, despite the promise of this approach, anion-templation strategies remain underdeveloped and examples of catenane-based host systems that are capable of optically or electrochemically sensing the presence of an anionic guest species are rare.^16^

Herein we describe the synthesis and solid-state structure of a new strapped-porphyrin-containing [2]catenane anion host system, which is assembled by using chloride anion templation in combination with aromatic donor–acceptor interactions and pyridine–zinc ligation.^17^ After removal of the templating anion, the catenane’s halide anion-recognition and sensing properties were probed by ^1^H NMR, UV/Vis and fluorescence titration experiments.

## Results and Discussion

### Design and synthetic strategy

**Figure 1:**
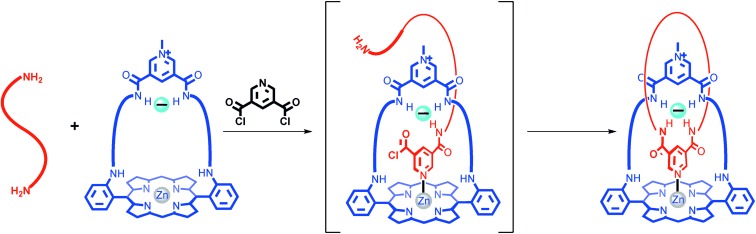
Cartoon representation of the intended synthetic route to the target strapped-porphyrin-containing [2]catenane, which incorporates an intercomponent pyridine–zinc coordinative bond.

We have previously employed a chloride-anion-templated, amide-condensation-based clipping strategy to assemble [2]catenane architectures in which bidentate hydrogen-bond-donor groups from each of the interlocked macrocyclic components converge towards a central, three-dimensional binding cavity, where the halide anion template is encapsulated. Upon removal of the template, the [2]catenanes were shown to recognise halide anions selectively over oxoanions in 1:1 CDCl_3_/CD_3_OD, which is primarily attributed to the optimal size- and shape-complementarity between the halide anions and the hosts’ interlocked binding domains.b13d In the current study we have modified our previous catenane design by incorporating a zinc(II) metalloporphyrin unit into one of the interlocked macrocyclic components, and a 3,5-pyridine bis(amide) motif into the second macrocycle component, in order to introduce an intercomponent pyridine–zinc interaction into the final interlocked structure (Figure 1).

We anticipated that this coordinative interaction could be exploited in several ways: as well as providing an auxiliary templating interaction during catenane assembly, the pyridine–zinc ligation process was predicted to enhance the anion recognition properties of the [2]catenane host system by increasing both the overall preorganisation of the host system and the acidity of the 3,5-pyridine bis(amide) hydrogen-bond-donor groups; it was also envisaged that the pyridine–zinc bond would create a direct through-bond communication pathway between the anion recognition site and the porphyrin chromophore, which could potentially enable the [2]catenane host system to optically sense the presence of an encapsulated guest anion.

### Synthesis of pyridinium-strapped porphyrin macrocycle

**Scheme1:**
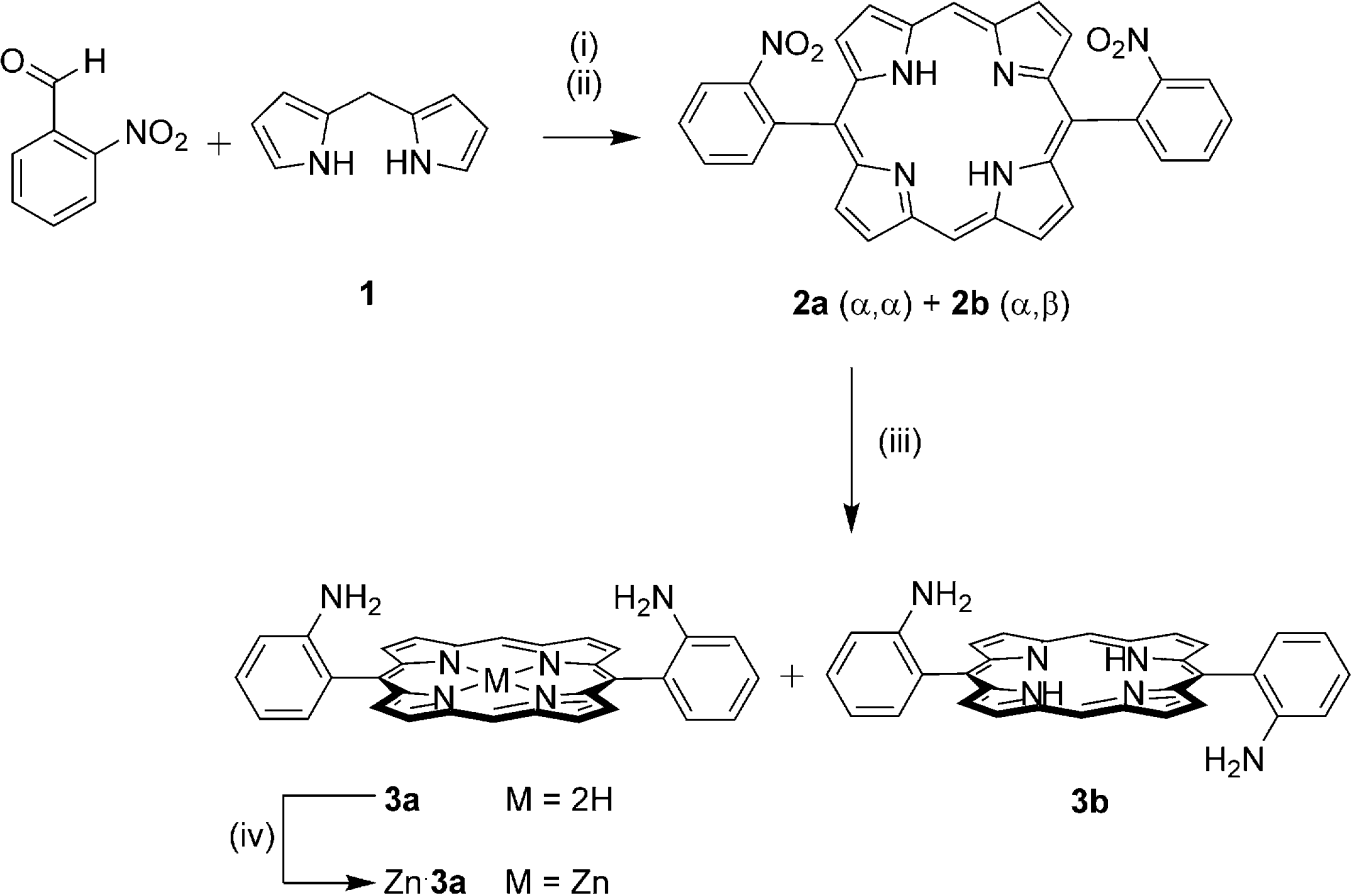
Synthesis of the bis(amino)porphyrin 3. Reagents and conditions: i) BF_3_⋅OEt_2_, EtOH, CH_2_Cl_2_, RT, 1 h; ii) *p*-chloranil, reflux, 1 h, 25 %; iii) SnCl_2_⋅2 H_2_O, 37% HCl_(aq)_, RT, 16 h, 37 % (3 a) and 40 % (3 b); iv) Zn(OAc)_2_⋅2 H_2_O, CH_2_Cl_2_, MeOH, RT, 18 h, 91 %.

**Scheme2:**
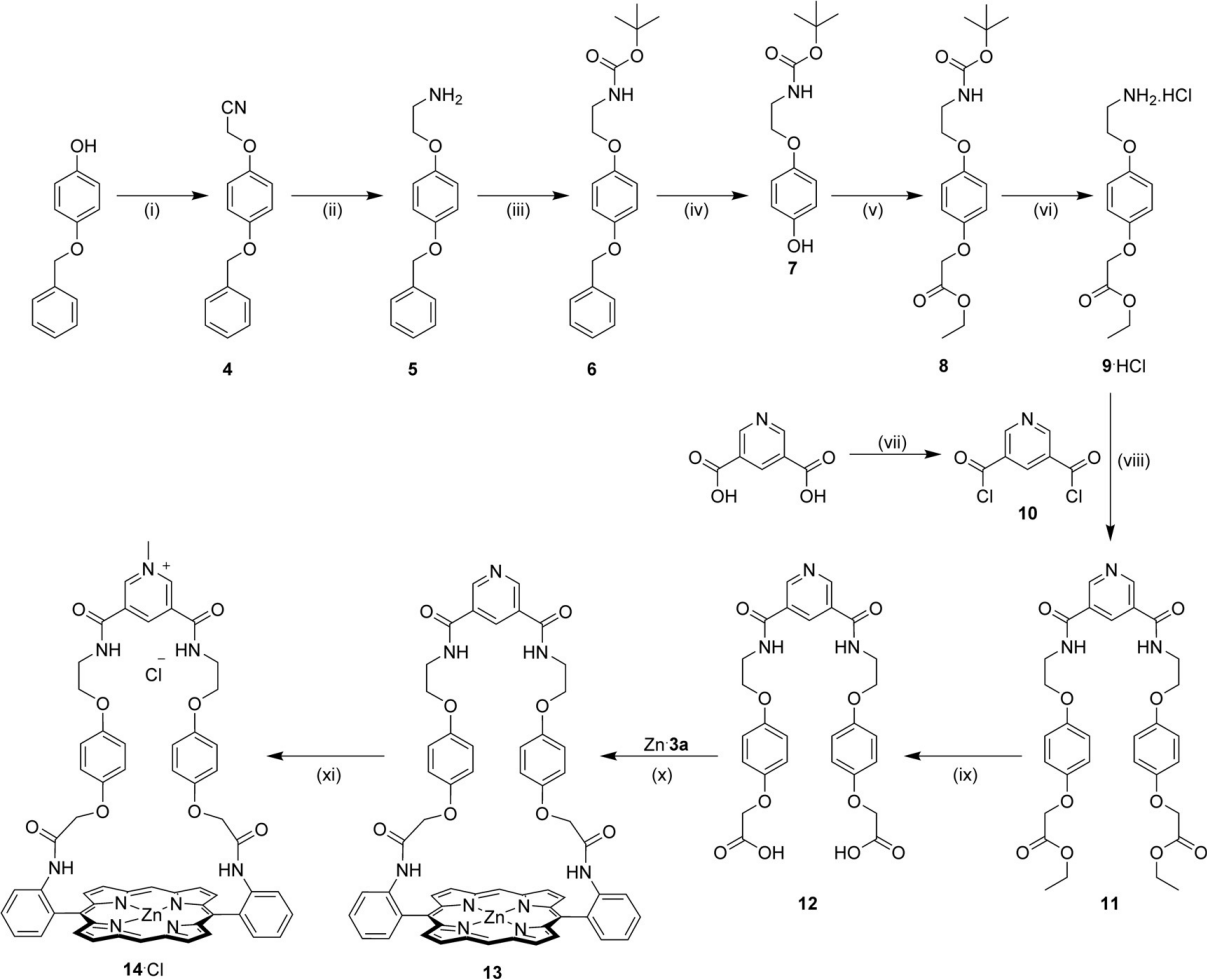
Synthesis of the pyridinium-strapped macrocycle 14⋅Cl. Reagents and conditions: i) Bromoacetonitrile, K_2_CO_3_, acetone, reflux, 18 h, 97 %; ii) LiAlH_4_, Et_2_O, reflux, 84 %; iii) Boc_2_O, Et_3_N, CH_2_Cl_2_, RT, 87 %; iv) H_2_, Pd/C, CHCl_3_, MeOH, RT, 95 %; v) ethylbromoacetate, NaH, THF, RT, 1 h, 50 °C, 12 h, 94 %; vi) HCl_(g)_, Et_2_O, RT, 97 %; vii) (COCl)_2_, DMF (cat.), CH_2_Cl_2_, RT, quantitative yield; viii) Et_3_N, CH_2_Cl_2_, 0 °C to RT, 18 h, 73 %; ix) KOH, CH_2_Cl_2_, MeOH, H_2_O RT, 88 %; x) EDC⋅HCl, HOBt, DMAP, DMF, RT, 44 %; xi) MeI, NaHCO_3_, DMF, 80 °C, 2 h, then NH_4_Cl_(aq)_, 99 %.

The synthesis of the new pyridinium bis(amide)- strapped porphyrin macrocyclic fragment of the target [2]catenane was carried out as outlined in Schemes 1 and 2. Initially the bis(amino)porphyrin precursor Zn⋅**3 a** was prepared (Scheme 1). The *trans*-substituted dinitroporphyrin **2**^18^ was obtained from a BF_3_-catalysed [2+2] condensation reaction between *meso*-unsubstituted dipyrromethane **2**^19^ and 2-nitrobenzaldehyde, with subsequent *p*-chloranil oxidation.^20^ Compound **2** was obtained in 25 % yield, as an approximately equimolar mixture of the α,α- and α,β-atropisomeric forms **2 a** and **2 b**. Reduction of this isomeric mixture with SnCl_2_/HCl afforded the corresponding α,α*-* and α,β-bis(amino)porphyrin atropisomers **3 a** and **3 b**, which were separated by column chromatography.^21^ On the basis of molecular dipole considerations, and by analogy with related compounds in the literature,^22^ the more polar compound was assigned as the desired α,α-isomer **3 a**, which was isolated in 37 % yield. The α,β-isomer **3 b** was isolated in a comparable yield of 40 %.^23^ Metallation of the α,α-bis(amine) **3 a** by treatment with excess Zn(OAc)_2_⋅2 H_2_O afforded the corresponding zinc(II) metalloporphyrin Zn⋅**3 a** in 91 % yield.

**Figure 2:**
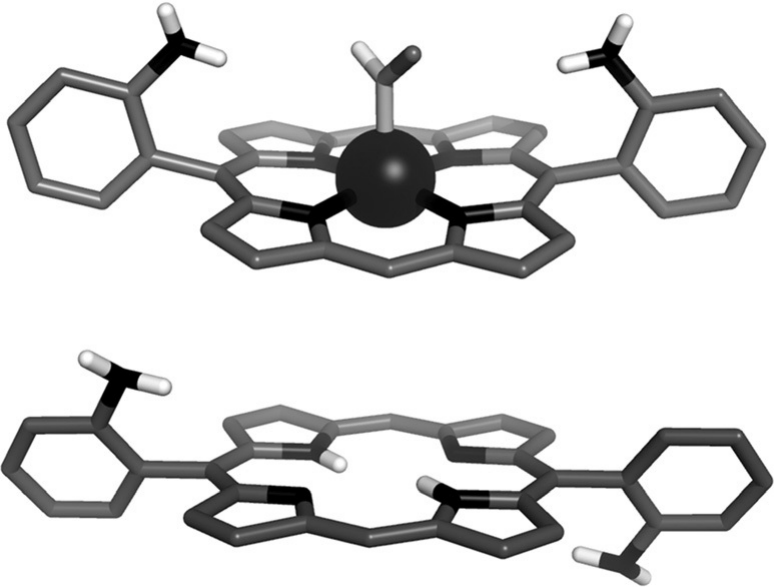
Solid-state structures of the α,α- and α,β-aminoporphyrin derivatives Zn(MeOH)⋅3 a (top) and 3 b (bottom). For clarity, nonpolar hydrogen atoms have been omitted and only one of the two fractional occupancies of the axially coordinated methanol solvate molecule is shown.

The assignment of the atropisomers was confirmed by solid-state structural characterisation of compounds Zn⋅**3 a** and **3 b** (Figure 2). In both structures, the *meso*-aryl substituents are arranged almost orthogonally to the planes of the porphyrins (mean dihedral angle=86.7° and 67.8° for compounds Zn⋅**3 a** and **3 b** respectively). For compound Zn⋅**3 a**, the desired *cis* arrangement of the amino groups is observed, whereas for compound **3 b** the *meso*-substituents adopt a *trans* conformation, with the two amino groups diverging away from the porphyrin plane. For both compounds, the hybridization states of the aniline nitrogen atoms appear to be intermediate between sp^3^ and sp^2^, with all of the aniline nitrogen torsion angles falling within the range 150–165°.^24^ The structure of the metalloporphyrin Zn⋅**3 a** confirms that the zinc(II) cation adopts the expected five-coordinate square pyramidal coordination environment in the solid state, with an axially coordinated methanol molecule occupying the apical position of the square pyramid (mean Zn–N distance: 2.048(2) Å; Zn–O distance: 2.166(20) Å).^25^ The zinc(II) cation is located 0.174 Å above the mean plane of the porphyrin, which is significantly ruffled, in contrast to the highly planar free base porphyrin derivative **3 b.**

Having isolated the α,α-bis(amino)porphyrin precursor Zn⋅**3 a**, the strap component of the porphyrin-containing macrocycle was constructed in ten steps from commercially available 4-(benzyloxy)phenol. Reaction of this precursor with bromooacetonitrile, followed by cyano-group reduction, Boc-protection of the amino group and hydrogenative debenzylation afforded the phenol derivative **7**,b13a which was condensed with ethyl bromoacetate to provide compound **8**. Cleavage of the *N*-Boc protecting group by bubbling HCl_(g)_ through a solution of compound **8** in Et_2_O yielded the corresponding amine as its hydrochloride salt, **9**⋅HCl. This was condensed with 0.5 equivalents of the bis-acid chloride **10** to produce the bis-ester intermediate **11**, which was subsequently converted into the bis-acid-functionalised strap precursor **12** in 88 % yield by base-mediated hydrolysis of the ester groups. An EDC-promoted coupling reaction [EDC=*N*-(3-dimethylaminopropyl)-*N*′-ethylcarbodiimide, which was added to the reaction as a hydrochloride salt] between this bis-acid derivative and the α,α-bis(amino)porphyrin Zn⋅**3 a** in DMF afforded the pyridine-strapped macrocycle **13** in 44 % yield.^26^ Finally, alkylation of the pyridine group by treatment of macrocycle **13** with MeI in the presence of NaHCO_3_^27^ in DMF, followed by repeated extraction with NH_4_Cl_(aq)_, afforded the chloride salt of the target pyridinium-strapped porphyrin macrocycle, **14**⋅Cl, in 99 % yield (Scheme 2).

**Figure 3:**
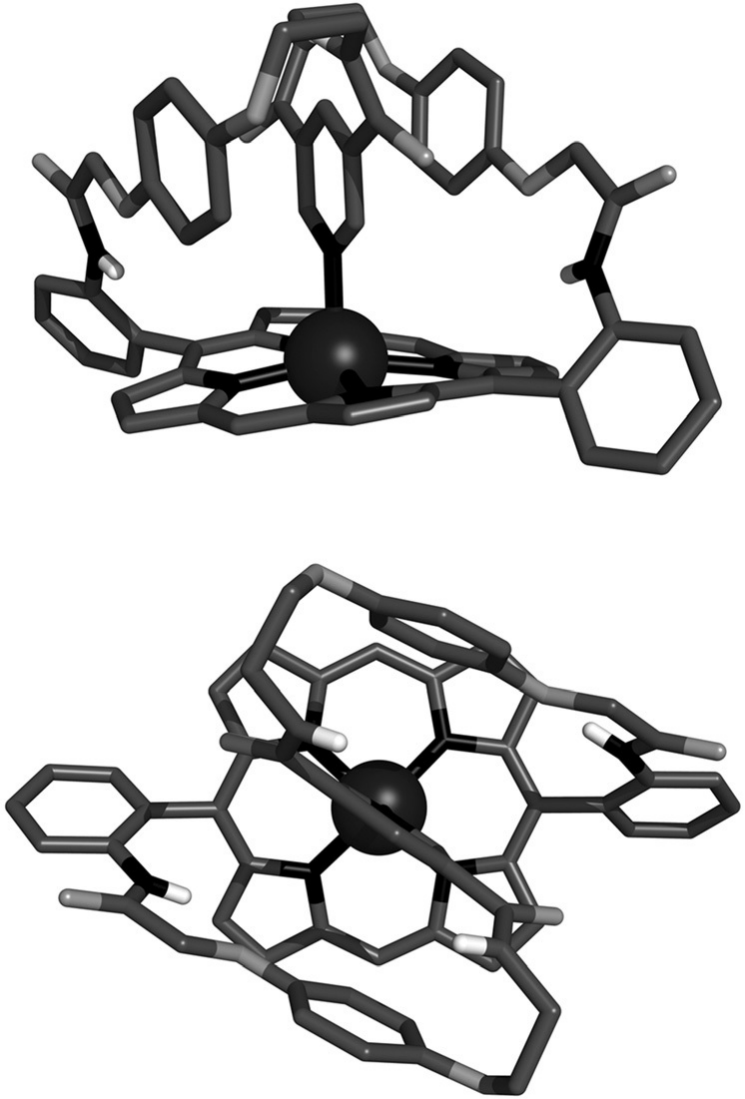
Orthogonal views of the solid-state structure of the pyridine- strapped porphyrin macrocycle 13. Solvent molecules and nonpolar hydrogen atoms have been omitted for clarity.

**Figure 4:**
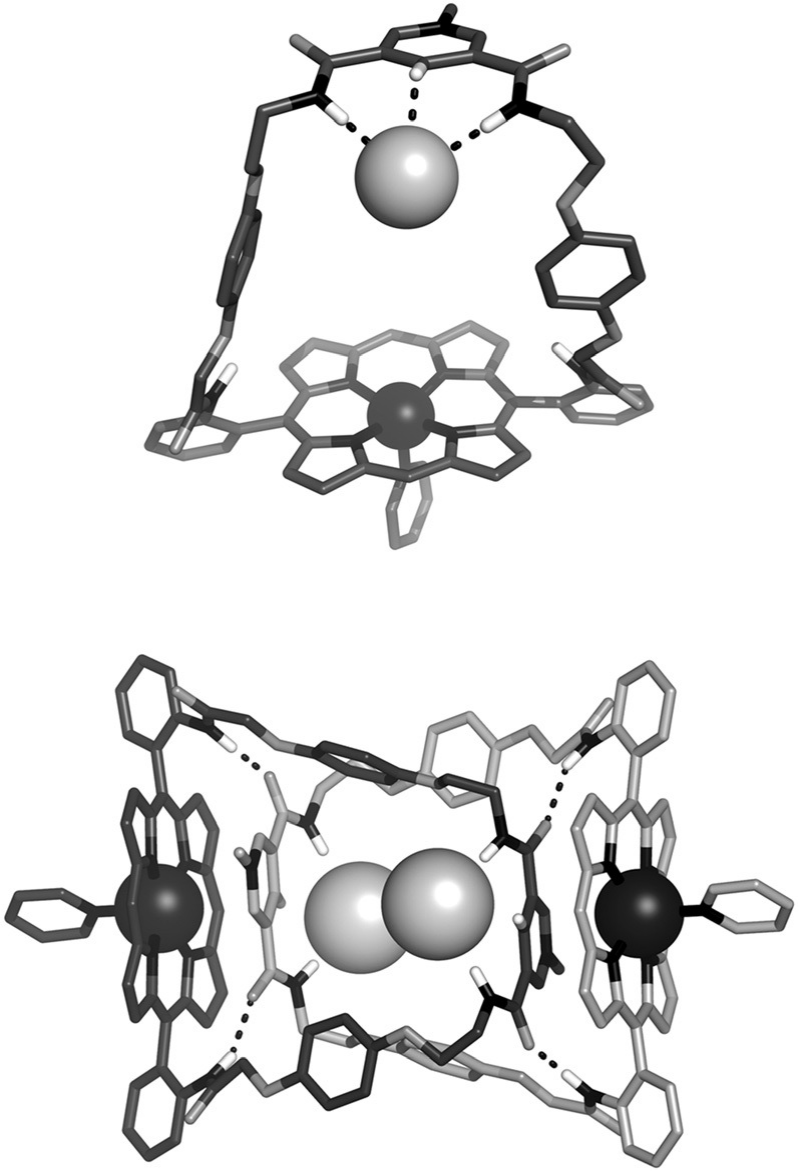
Solid-state structure of the pyridine solvate of the pyridinium-strapped porphyrin macrocycle 14⋅Cl: content of the asymmetric unit (top) and view of the head-to-tail dimer which forms as a result of crystal packing (bottom). Nonpolar hydrogen atoms have been omitted for clarity. Hydrogen bonds are represented as dashed lines.

Both of the new macrocycles **13** and **14**⋅Cl were characterised by X-ray crystallography in the solid state. The crystal structure of the pyridine-strapped macrocycle **13** (Figure 3) reveals the existence of an intramolecular coordinative interaction between the pyridyl nitrogen atom and the zinc(II) cation (Zn–N_pyr_ distance: 2.222(3) Å), which causes the strap to fold inwards, inducing a parallel stacking arrangement between the 1,4-hydroquinone and 3,5-pyridine bis(amide) moieties. A saddle distortion of the porphyrin unit is also apparent. For the *N*-methylpyridinium-strapped macrocycle **14**⋅Cl, it is not possible for an analogous intramolecular pyridine–zinc interaction to occur, and the axial coordination site is instead occupied by a pyridine solvate molecule, which ligates to the outer face of the porphyrin unit (Zn–N_pyr_ distance: 2.136(4) Å; Figure 4). The macrocycle’s 3,5-pyridinium bis(amide) group adopts a *syn*–*syn* conformation and the chloride counteranion is held within the resultant binding cleft by three short NH⋅⋅⋅Cl and CH⋅⋅⋅Cl contacts (N⋅⋅⋅Cl distances: 3.348(1) and 3.233(1) Å; C⋅⋅⋅Cl distance: 3.231(2) Å). Examination of the crystal packing reveals that the pyridinium macrocycle **14**⋅Cl forms a head-to-tail dimer in the solid state. Each dimeric unit appears to be stabilised by two complementary intermolecular aromatic stacking interactions between the pyridinium and porphyrin groups, in addition to four NH⋅⋅⋅O amide–amide hydrogen bonds.

### Synthesis of strapped-porphyrin-containing [2]catenane

**Scheme3:**
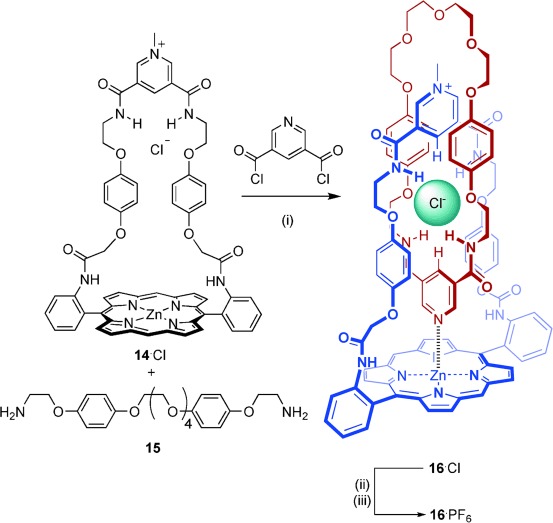
Synthesis of the [2]catenanes 16⋅Cl and 16⋅PF_6_. Reagents and conditions: i) Et_3_N, CH_2_Cl_2_, RT, 2 h, 30 %; ii) AgNO_3_, DMSO/H_2_O, RT, 45 min; iii) NH_4_PF_6(aq)_, 73 %.

Condensation of the bis(amine) derivative **15**^28^ with 3,5-bis(chlorocarbonyl) pyridine **10** in the presence of the pyridinium-strapped porphyrin macrocycle **14**⋅Cl in CH_2_Cl_2_ afforded the [2]catenane **16**⋅Cl in 30 % yield after purification by preparative TLC and recrystallisation (Scheme 3). The chloride anion template was removed by stirring compound **16**⋅Cl with AgNO_3_ in 95:5 DMSO/H_2_O, before precipitation of the hexafluorophosphate salt **16**⋅PF_6_ in 73 % yield by addition of NH_4_PF_6(aq)_.

### [2]Catenane characterisation

**Figure 5:**
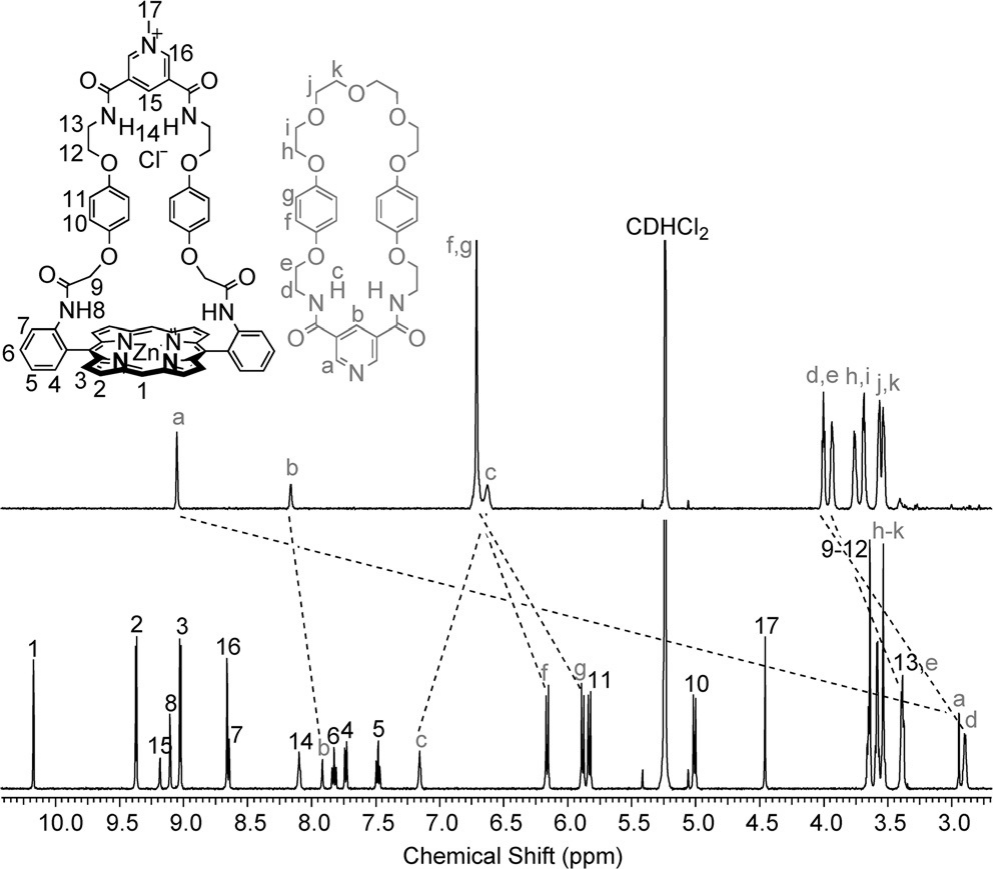
Partial ^1^H NMR spectra (500 MHz) of macrocycle 17 (top spectrum) and the [2]catenane 16⋅Cl (bottom spectrum) in CD_2_Cl_2_ at 293 K.


The [2]catenanes **16**⋅Cl and **16**⋅PF_6_ were fully characterised by electrospray mass spectrometry and ^1^H and ^13^C NMR spectroscopy techniques in solution, and by X-ray crystallography in the solid state. The ^1^H NMR spectrum of the [2]catenane **16**⋅Cl in CD_2_Cl_2_ is compared with that of non-interlocked 3,5-pyridine bis(amide)-functionalised macrocycle **17**^29^ in Figure 5.

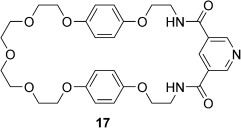


A dramatic >6 ppm upfield shift in the signal for the aromatic *ortho* pyridine proton a is observed upon incorporation of macrocycle **17** into the [2]catenane, with concomitant 0.3–1.2 ppm upfield shifts in the signals corresponding to the *para* pyridine proton b and aliphatic CH_2_ protons d and e. This indicates that the pyridyl group is located within the shielding region created by the porphyrin ring currents, and therefore strongly suggests that the [2]catenane is stabilised by an intercomponent pyridine–zinc interaction in solution. In contrast, the resonance for amide proton c shifts downfield, which is consistent with the pyridine bis(amide) group participating in NH⋅⋅⋅Cl hydrogen-bonding interactions with the chloride anion, since these interactions would be expected to polarise the amide NH bonds. The pronounced upfield shift and splitting of the resonances for hydroquinone protons f and g is diagnostic of secondary aromatic-donor–acceptor interactions between the electron-rich hydroquinone groups in the neutral macrocycle and the electron-deficient pyridinium component of the charged macrocycle.^30^ In addition, a number of through-space interactions between the two interlocked macrocyclic components of the catenane were observed by 2D ^1^H NMR ROESY spectroscopy in CD_2_Cl_2_ and [D_6_]DMSO, which provided further supportive evidence for the interlocked nature and proposed solution conformation of the [2]catenane (see the Supporting Information, Figures S20 and S24).

Single crystals of the [2]catenanes **16**⋅Cl and **16**⋅PF_6_ that were suitable for X-ray structural determination were grown by layered diffusion of hexane into a CH_2_Cl_2_/MeOH solution of the chloride salt **16**⋅Cl, and by layered diffusion of diisopropyl ether into an acetone solution of the hexafluorophosphate salt **16**⋅PF_6_. In both cases, the crystals were small and weakly diffracting, and X-ray diffraction data were collected by using synchrotron radiation.

**Figure 6:**
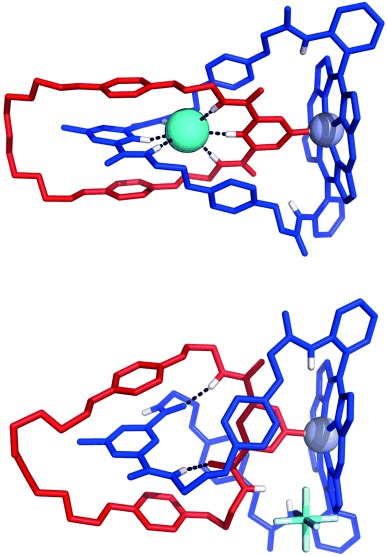
Solid-state structures of the [2]catenane 16⋅Cl (top) and the [2]catenane 16⋅PF_6_ (bottom). Solvent molecules and nonpolar hydrogen atoms have been omitted for clarity. Hydrogen bonds are represented as dashed lines.

The crystal structure of the chloride-complexed catenane **16**⋅Cl (Figure 6) confirms the existence of an intercomponent pyridine–zinc coordinative bond in the solid state (Zn–N_pyr_ distance: 2.138(2) Å). The chloride anion is encapsulated within the pseudo-octahedral interlocked binding cavity defined by the orthogonally disposed 3,5-pyridinium bis(amide) and 3,5-pyridine bis(amide) moieties. The hydrogen atoms from each of the six aromatic CH and amide NH hydrogen-bond-donor groups are directed towards the encapsulated chloride anion, and six short X⋅⋅⋅Cl (X=C, N) contacts are observed, with X⋅⋅⋅Cl distances ranging from 3.285 to 3.353(2) Å and X–H⋅⋅⋅Cl angles ranging from 159 to 180°. A parallel donor–acceptor–donor aromatic stacking arrangement between the electron-rich 1,4- hydroquinone and electron-deficient pyridinium motifs is also observed, with centroid-to-centroid distances of 3.493 and 3.822 Å.

The diffraction data obtained for the hexafluorophosphate catenane **16**⋅PF_6_ were unusually weak and the structure was found to incorporate a significant degree of disorder, necessitating the use of extensive restraints during minimisation. Nevertheless, it is evident from the structure that the conformation of the hexafluorophosphate catenane **16**⋅PF_6_ is largely unchanged from that of the chloride catenane **16**⋅Cl in the solid state (Figure 6). In the absence of an encapsulated chloride anion, the pyridine–zinc coordinative bond and approximate interlocked co-conformation of the two macrocycles are preserved, but the 3,5-pyridinium bis(amide) and 3,5-pyridine bis(amide) groups adopt a *syn*–*anti* conformation, which is stabilised by two intercomponent NH⋅⋅⋅O amide–amide hydrogen bonds. The hexafluorophosphate counteranion is not involved in short contacts with the hydrogen-bond-donor groups from either of the bis(amide) motifs, which is in accordance with its assumed non-coordinating role.^31^

### Halide recognition and sensing experiments

Encouraged by the crystallographic evidence that the chloride counteranion is encapsulated within the interlocked binding cavity of the [2]catenane **16**⋅Cl in the solid state, we employed ^1^H NMR, UV/Vis and fluorescence spectroscopic titration experiments to investigate the ability of the [2]catenane host system to recognise and sense halide anions in solution.

#### ^1^H NMR titration experiments

**Figure 7:**
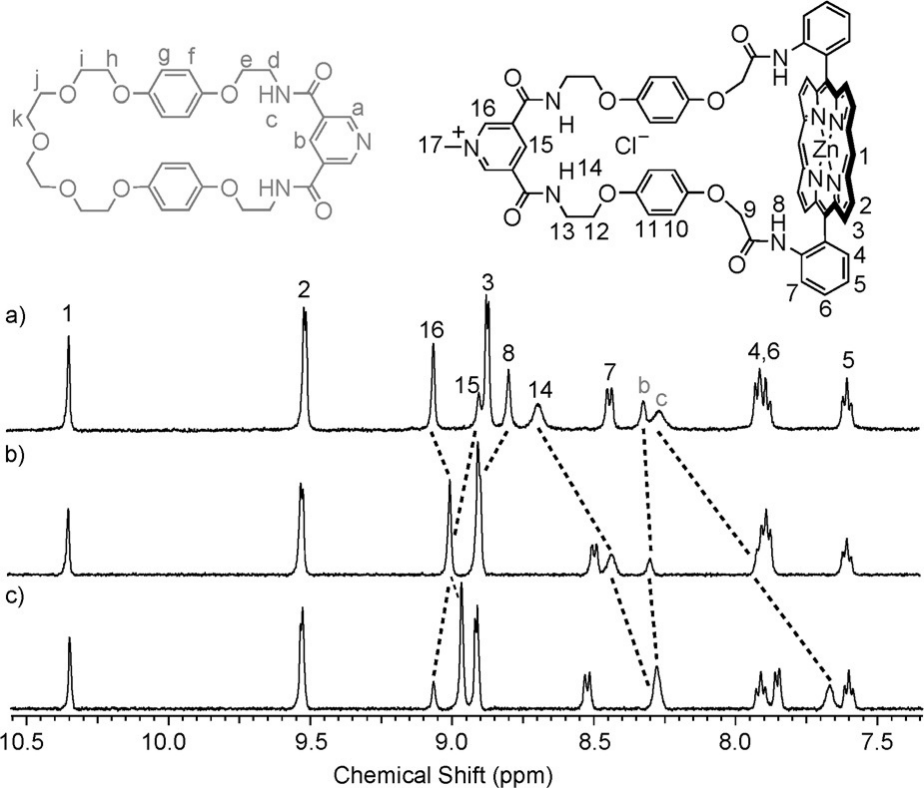
Partial ^1^H NMR spectra of a 1.5 mM solution of the [2]catenane 16⋅PF_6_ in [D_6_]DMSO at 293 K after addition of a) 0, b) 1 and c) 5 equivalents of TBACl.

**Figure 8:**
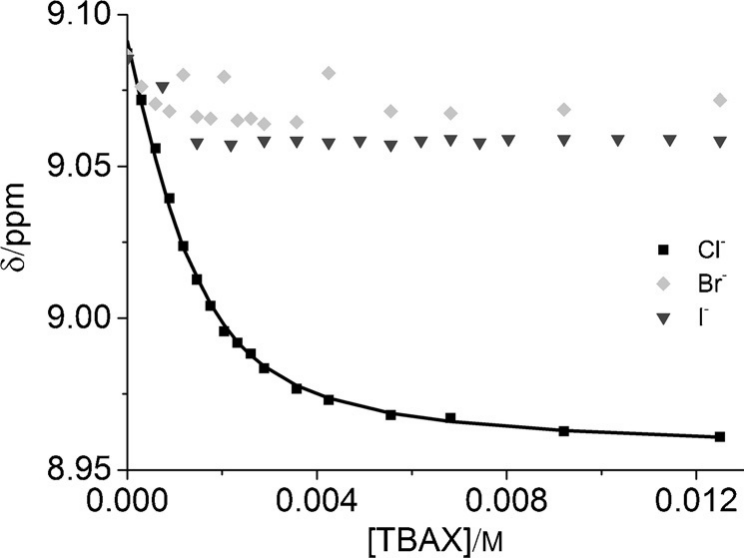
Changes in the chemical shift of the pyridinium proton 16 on addition of the TBAX salts (X=Cl^−^, Br^−^ and I^−^) to 1.5 mM solutions of compound 16⋅PF_6_ in [D_6_]DMSO at 298 K. Square data points represent experimental data; continuous line represents the calculated binding curve for *K*=2144 M^−1^.

Addition of an increasing concentration of tetrabutylammonium (TBA) chloride to a 1.5 mM solution of compound **16**⋅PF_6_ in [D_6_]DMSO induced progressive shifts in the ^1^H NMR signals for protons 14, 15 and c (Figure 7), which is consistent with fast-exchange complexation of the chloride anion within the [2]catenane’s interlocked binding domain. A 1:1 stoichiometric association constant of *K*=2144(149) M^−1^ was determined by analysis of the chloride-concentration-dependent shifts of the external pyridinium proton 16 with WinEQNMR2^32^ software (Figure 8). In contrast, comparable titration experiments using TBAI and TBABr salts produced no convincing evidence of a binding interaction between the larger halide anions and the catenane’s interlocked cavity. Addition of up to 10 equivalents of TBABr and TBAI to a [D_6_]DMSO solution of the catenane resulted in only slight (Δ*δ*≤0.11 ppm) perturbations in the ^1^H NMR signals for the cavity protons 14, 15 and c (Figure 8), which could not be definitively assigned to a binding event. The [2]catenane host system **16**⋅PF_6_ therefore appears to display an impressive selectivity for chloride over bromide and iodide anions in DMSO, which may reflect an optimal host–guest complementarity relationship between the chloride anion and the catenane’s preorganised hydrogen-bond- donating binding pocket.^33^

#### UV/Vis and fluorescence experiments

**Figure 9:**
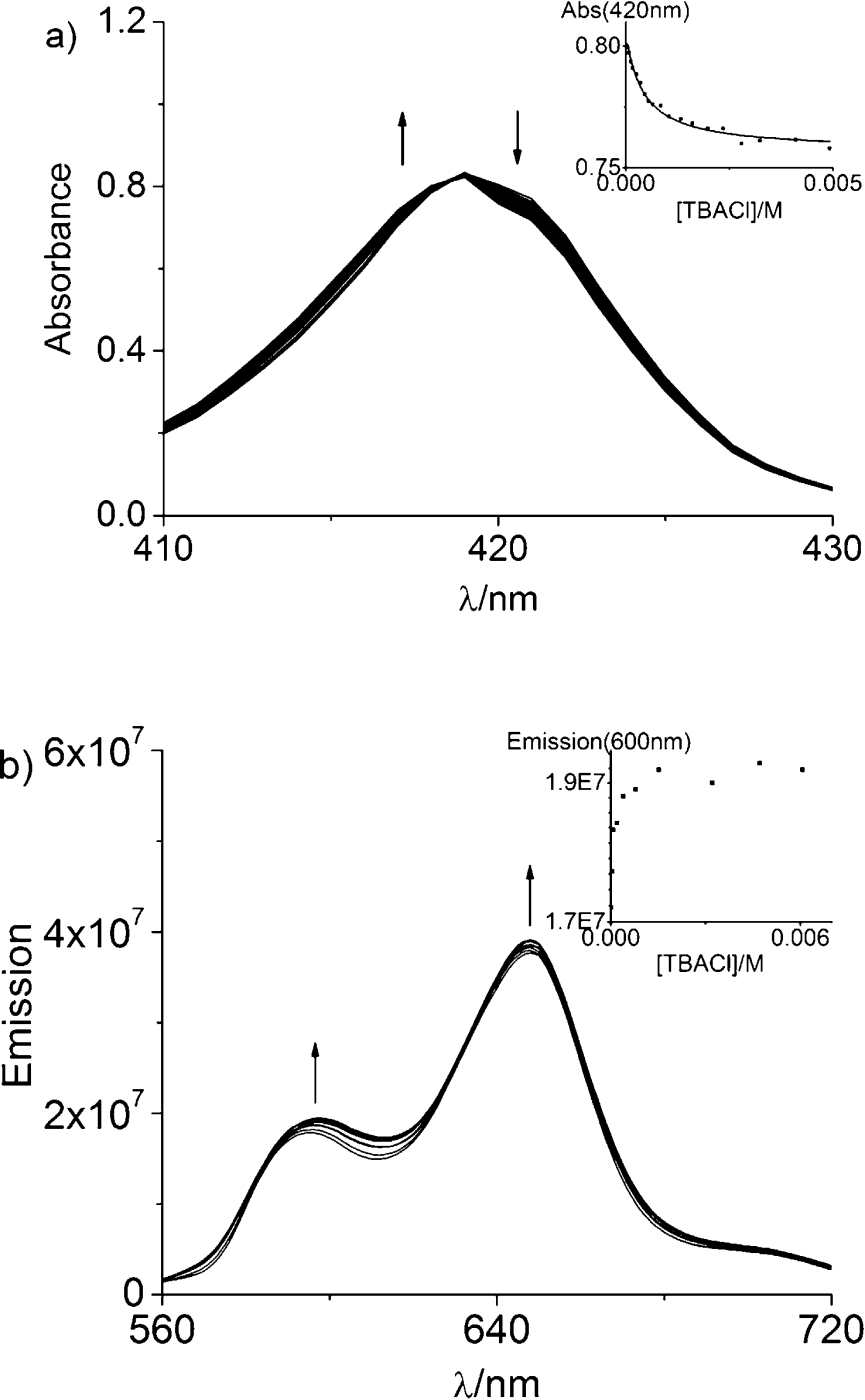
a) Changes in the Soret band absorbance of a 2 μM solution of the [2]catenane 16⋅PF_6_ in DMSO upon addition of an increasing concentration of TBACl (final TBACl concentration: 4.9 mM). Inset: change in absorbance at 420 nm as a function of [TBACl]; square data points represent experimental data; continuous line represents the calculated binding curve for *K*=2396 M^−1^. b) Changes in the fluorescence emission spectrum of a 40 μM solution of the the [2]catenane 16⋅PF_6_ in DMSO upon addition of an increasing concentration of TBACl (final TBACl concentration: 6.1 mM). *λ*_ex_=548 nm. Inset: change in emission at 600 nm as a function of [TBACl].

UV/Vis and fluorescence spectroscopic titration experiments revealed that the [2]catenane exhibits modest optical chloride-sensing capabilities: upon titration of TBACl into solutions of the catenane in DMSO a gradual hypsochromic shift of approximately 1 nm was observed in the Soret band absorbance, with the formation of a single isosbestic point at 418.5 nm,^34^ along with approximately 9 and 4 % increases in the intensities of the emission maxima at 595 nm and 650 nm, respectively (Figure 9). Analysis of the UV/Vis titration data by using Specfit^35^ software revealed a 1:1 stoichiometric association constant of log *K*=3.38±0.04 (*K*=2396 M^−1^), which is in good agreement with the value determined by ^1^H NMR spectroscopy. By comparison, addition of increasing concentrations of TBABr and TBAI to DMSO solutions of the catenane produced no discernible shifts in the maximum of the Soret band absorbance and only slight, random fluctuations in the intensities of the fluorescence emission spectra (see the Supporting Information, Figures S27 and S28), which corroborates the ^1^H NMR evidence that these larger halides do not significantly interact with the [2]catenane host system in DMSO.

## Conclusions

A new strapped-porphyrin-containing [2]catenane anion-host system was prepared through an amide-condensation-based clipping reaction, which was directed by chloride anion templation in combination with pyridine–zinc ligation and aromatic donor–acceptor interactions. Upon removal of the halide template, the catenane was shown to selectively recognise chloride (*K*=2144(149) M^−1^) over larger halide anions in the competitive solvent [D_6_]DMSO. The [2]catenane also exhibits a modest ability to optically sense chloride anions in DMSO through small but detectable changes in the absorption and emission spectra of the porphyrin chromophore.

## Experimental Section

All solvents and reagents were purchased from commercial suppliers and used as received, unless otherwise stated. Dry solvents were obtained by purging with N_2_ and then passing through an MBraun MPSP-800 column. H_2_O was deionised and microfiltered by using a Milli-Q Millipore machine. Et_3_N was distilled and stored over KOH. TBA salts were stored in a vacuum desiccator containing P_2_O_5_ prior to use. ^1^H, ^13^C, ^19^F and ^31^P NMR spectra were recorded on a Varian Mercury-VX 300, a Varian Unity Plus 500, a Bruker AVD500 or a Bruker AVII500 with cryoprobe at 293 K. Chemical shifts are quoted in parts per million relative to the residual solvent peak. Mass spectra were obtained by using a Micromass LCT (ESMS) instrument or a MALDI Micro MX instrument. Electronic absorption spectra were recorded on a PG instruments T60U spectrometer.

### X-ray crystallography

Single crystal diffraction data for compounds **3 b** and Zn⋅**3 a** were collected at 150(2) K using graphite monochromated Mo_Kα_ radiation (*λ*=0.71073 Å) on a Nonius Kappa CCD diffractometer. Cell parameter determination and refinement and raw frame data integration were carried out by using the DENZO-SMN package.^36^ Diffraction data for compounds **13**, **14**⋅Cl, **16**⋅Cl and **16**⋅PF_6_ were collected at 100(2) K by using silicon double crystal monochromated synchrotron radiation (*λ*=0.68890 Å) at Diamond Light Source, beamline I19,^37^ with a custom-built Rigaku diffractometer.^38^ Cell parameter determination and refinement and raw frame data integration were carried out by using the CrysAlisPro^39^ package. The structures were solved by charge-flipping methods using SUPERFLIP^40^ and refined by full matrix least squares on F^2^ using the CRYSTALS^41^ suite. All non-hydrogen atoms were refined with anisotropic displacement parameters. Where appropriate, disordered regions were modelled by using refined partial occupancies, geometric restraints were applied to ensure a physically reasonable model, and thermal and vibrational restraints were applied to maintain sensible ADPs; in addition, when present, diffuse disordered solvent and counteranions were modelled by treating the discrete Fourier transform of the void region as contributions to the calculated structure factors with PLATON/SQUEEZE.^42^ Hydrogen atoms were generally visible in the difference map and were treated in the conventional manner.^43^ CCDC 1412108–1412113 contain the supplementary crystallographic data (excluding structure factors) for this paper. These data are provided free of charge by The Cambridge Crystallographic Data Centre.

### Synthetic procedures and characterisation data

The syntheses of the known compounds **2**^18^ and **4–7**b13a, 44 are described in the supporting information. Compounds **1,**^19^ **15**^28^ and **17**^29^ were prepared by slight modification of previously reported procedures.

**α,α- and α,β*-*Bis(amino)porphyrins 3 a and 3 b**: 5,15-Bis-(2-nitrophenyl)porphyrin **2** (0.47 g, 0.84 mmol) was suspended in 37 % HCl_(aq)_ (35 mL). The mixture was sonicated briefly and then stirred at room temperature for 20 min. SnCl_2_⋅2 H_2_O (0.10 g, 4.42 mmol) was added and the reaction mixture was stirred vigorously under N_2_ for 16 h. After cooling to 0 °C, saturated NH_4_OH_(aq)_ was added cautiously until the pH reached 7. CH_2_Cl_2_ (30 mL) was added. The mixture was stirred vigorously at room temperature for 30 min and then transferred to a separating funnel. The layers were separated and the aqueous layer was extracted with CH_2_Cl_2_ (3×25 mL). The combined CH_2_Cl_2_ extracts were washed with H_2_O (2×50 mL), dried over MgSO_4_ and concentrated on a rotary evaporator to give a purple solid, which contained a mixture of the α,α- and α,β-bis(amino)porphyrin derivatives **3 a** and **3 b**. This atropisomeric mixture was separated by column chromatography. The α,β*-*isomer **3 b** was eluted with 98:2 CH_2_Cl_2_/EtOAc and obtained as a purple solid (0.165 g, 40 %). 9:1 CH_2_Cl_2_/EtOAc was then used to elute the α,α-isomer **3 a**, which was also obtained as a purple solid (0.152 g, 37 %).

**Characterisation data for α,α-5,15-bis(2-aminophenyl)porphyrin 3 a**: ^**1**^**H NMR** (500 MHz; CDCl_3_): *δ*=10.31 (s, 2 H, porphyrin-*meso*-H), 9.41 (d, ^3^*J*=4.6 Hz, 4 H, β-pyrrole-H), 9.12 (d, ^3^*J*=4.6 Hz, 4 H, β-pyrrole-H), 7.97 (d, ^3^*J*=8.2 Hz, 2 H, Ar–H), 7.67–7.64 (m, 2 H, Ar–H), 7.25–7.22, (m, 2 H, Ar–H), 7.17 (d, ^3^*J*=8.2 Hz, 2 H, Ar–H), 3.56 (br s, 4 H, NH_2_), −3.15 ppm (br s, 2 H, pyrrole-NH); ^13^C NMR (75 MHz; CDCl_3_): *δ*=147.3, 146.9, 145.6, 134.8, 132.0, 130.8, 129.7, 126.3, 117.7, 115.3, 114.9, 105.0 ppm; UV/vis (CH_2_Cl_2_): *λ*_max_ (*ε*)=405 (250 000), 501 (18 000), 536 (5000), 574 (6000), 628 nm (1500 mol^−1^ m^3^ cm^−1^); ESMS *m/z*: 493.21 ([*M*+H]^+^; C_32_H_25_N_6_ requires 493.21); HRMS *m/z*: 493.2133 ([*M*+H]^+^; C_32_H_25_N_6_ requires 493.2135).

**Characterisation data for α,β-5,15-bis(2-aminophenyl)porphyrin 3 b**: ^**1**^**H NMR** (500 MHz; CDCl_3_): *δ*=10.31 (s, 2 H, porphyrin-*meso*-H), 9.40 (d, ^3^*J*=3.8 Hz, 4 H, β-pyrrole-H), 9.12 (d, ^3^*J*=3.8 Hz, 4 H, β-pyrrole-H), 7.92 (d, ^3^*J*=7.6 Hz, 2 H, Ar–H), 7.67–7.64 (m, 2 H, Ar–H), 7.24–7.21, (m, 2 H, Ar–H), 7.18 (d, ^3^*J*=8.2 Hz, 2 H, Ar–H), 3.59 (br s, 4 H, NH_2_), −3.16 ppm (br s, 2 H, pyrrole-NH); ESMS *m/z*: 493.22 ([*M*+H]^+^; C_32_H_25_N_6_ requires 493.21; UV/vis (CH_2_Cl_2_): *λ*_max_ (*ε*)=405 (268 000), 502 (19 000), 536 (5500), 574 (6000), 629 nm (1000 mol^−1^ m^3^ cm^−1^); HRMS *m/z*: 493.2134 ([*M*+H]^+^; C_32_H_25_N_6_ requires 493.2135). Single crystals suitable for X-ray structural determination were grown by slow evaporation from CH_2_Cl_2_. Single crystal data: C_32_H_24_N_6_, *M*r=492.57; monoclinic, *P*2_1_/*c*; *a*=8.5216(2), *b*=10.9750(3), *c*=13.7764(4) Å; *α*=*γ*=90°, *β*=102.6958(14)°; *V*=1256.96(6) Å^3^; data/restraints/parameters: 2399/0/172; *R*_int_=0.024; final *R*_1_=0.047 (*I*>2*σ*(*I*)); *wR*_2_=0.118 (*I*>2*σ*(*I*)); Δρ_min_,_max_=−0.24, +0.35 e Å^−3^.

**Zn^II^ α,α-bis(amino)porphyrin Zn⋅3 a**: The α,α-bis(amino)porphyrin **3 a** (0.15 g, 0.30 mmol) was dissolved in CH_2_Cl_2_ (25 mL) and a solution of Zn(OAc)_2_⋅2H_2_O (0.33 g, 1.52 mmol) in MeOH (25 mL) was added. The solution was stirred at room temperature under nitrogen for 18 h, before being concentrated on a rotary evaporator without applying heat. The residual solid was dissolved in DMF (2 mL) and H_2_O (40 mL) was added. The resulting precipitate was collected by filtration, washed with H_2_O (8×15 mL) followed by MeOH (2×2.5 mL) and dried under high vacuum. After recrystallization (CH_2_Cl_2_/hexane), the product was obtained as a pink/purple solid (0.15 g, 91 %). ^1^H NMR (300 MHz; [D_6_]DMSO): *δ*=10.29 (s, 2 H, *meso*-H), 9.46 (d, ^3^*J*=4.7 Hz, 4 H, β*-*pyrrole-H), 8.93 (d, ^3^*J*=4.7 Hz, 4 H, β*-*pyrrole-H), 7.77 (dd, ^3^*J*=7.3 Hz, ^4^*J*=1.5 Hz, 2 H, *meso*-Ar–H), 7.58–7.53 (m, 2 H, *meso*-Ar–H), 7.16 (d, ^3^*J*=7.6 Hz, *meso*-Ar–H), 7.09–7.04 (m, 2 H, *meso*-Ar–H), 4.36 ppm (s, 4 H, NH_2_); ^13^C NMR (75 MHz; [D_6_]DMSO): *δ*=149.5, 149.0, 148.2, 148.1, 134.3, 132.2, 131.4, 129.0, 126.7, 115.6, 115.4, 114.6, 105.6 ppm; ESMS *m/z*: 555.15 ([*M*+H]^+^; C_32_H_23_N_6_Zn requires 555.13); HRMS *m/z* 555.1280 ([*M*+H]^+^; C_32_H_23_N_6_Zn requires 555.1270); UV/vis (CH_2_Cl_2_): *λ*_max_ (ε)=406 (107 000), 535 (7000), 570 nm (2000 mol^−1^ m^3^ cm^−1^). Single crystals of the methanol solvate suitable for X-ray structural determination were grown by slow evaporation from CH_2_Cl_2_/MeOH. Single crystal data: C_33_H_26_N_6_OZn, *M*r=587.99; triclinic, *P*$\bar 1$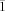, *a*=10.3352(2), *b*=11.5808(2), *c*=12.3227(3) Å; *α*=75.5368(8), *β*=69.8504(9), *γ*=88.4691(10)°; *V*=1337.91(5) Å^3^; data/restraints/parameters: 5470/0/388; *R*_int_=0.022; final *R*_1_=0.032 (*I*>2*σ*(*I*)); *wR*_2_=0.066 (*I*>2*σ*(*I*)); Δρ_min_,_max_=−0.52, +0.50 e Å^−3^.

**Ethyl 2-(4-{2-[(*tert*-butoxycarbonyl)amino]ethoxy}phenoxy)acetate 8**: *tert*-Butyl[2-(4-hydroxyphenoxy)ethyl]carbamate **7** (0.75 g, 2.96 mmol) was dissolved in dry THF (100 mL) and NaH (0.148 g of a 60 % dispersion in mineral oil, 3.70 mmol) was added. The mixture was stirred at room temperature under N_2_ for 20 min. Ethyl bromoacetate (0.99 g, 0.66 mL, 5.92 mmol) was added and the reaction mixture was heated to 50 °C, and maintained at this temperature under N_2_ for 18 h, before being cooled to room temperature, diluted with H_2_O (30 mL) and extracted with CH_2_Cl_2_ (4×30 mL). The combined organic extracts were washed with brine (50 mL), dried over MgSO_4_ and concentrated under reduced pressure. Purification of the residue by column chromatography (2 % MeOH in CH_2_Cl_2_) afforded the product as a viscous, pale yellow oil (0.95 g, 94 %). ^1^H NMR (300 MHz; [D_6_]acetone): *δ*=6.87–6.83 (m, 8 H, hydroquinone-Ar–H), 6.14 (br s, 1 H, NH), 4.63 (s, 2 H, OC*H*_2_CO_2_Et), 4.19 (quartet, ^3^*J*=7.0 Hz, 2 H, C*H*_2_CH_3_), 3.98 (t, ^3^*J*=5.58 Hz, 2 H, OC*H*_2_CH_2_NH) 3.45–3.39 (m, 2 H, OCH_2_C*H*_2_NH), 1.40 (s, 9 H, *t*Bu-CH_3_), 1.24 ppm (t, ^3^*J*=7.0 Hz, 3 H, CH_2_C*H*_3_); ^13^C NMR (75 MHz; [D_6_]acetone): *δ*=169.7, 156.8, 154.5, 153.4, 116.5, 116.2, 78.8, 68.2, 66.5, 61.4, 40.8, 28.7, 14.5 ppm; ESMS *m/z*: 362.15 ([*M*+Na]^+^; C_17_H_25_NNaO_6_ requires 362.16); 701.32 ([2 *M*+Na]^+^; C_34_H_50_N_2_NaO_12_ requires 701.33); HRMS *m/z*: 362.1569. ([*M*+Na]^+^; C_17_H_25_NNaO_6_ requires 362.1572).

**Ethyl 2-[4-(2-aminoethoxy)phenoxy]acetate hydrochloride 9⋅HCl**: Ethyl 2-(4-{2-[(*tert*-butoxycarbonyl)amino]ethoxy}phenoxy)acetate **8** was dissolved in Et_2_O (25 mL). Gaseous HCl was bubbled slowly through the solution for a period of 2 h, during which time a white precipitate formed. The mixture was then stirred at room temperature under N_2_ for an additional 2 h. The precipitate was collected by filtration, washed with Et_2_O (5×5 mL) and dried under vacuum to afford the product as a white solid (0.75 g, 97 %). ^1^H NMR (300 MHz; [D_6_]DMSO): *δ*=8.12 (br s, 3 H, NH_3_⋅Cl), 6.94–6.87 (m, 4 H, hydroquinone-Ar–H), 4.71 (s, 2 H, OC*H*_2_CO_2_Et), 4.15 (quartet, ^3^*J*=7.0 Hz, 2 H, C*H*_2_CH_3_), 4.10 (t, ^3^*J*=5.3 Hz, 2 H, OC*H*_2_CH_2_NH), 3.19–3.15 (br m, 2 H, OCH_2_C*H*_2_NH), 1.20 ppm (t, ^3^*J*=7.0 Hz, 3 H, CH_2_C*H*_3_); ^13^C NMR (75 MHz; [D_6_]DMSO): *δ*=168.9, 152.4, 152.1, 115.7, 115.5, 65.2, 64.8, 60.6, 38.3, 14.1 ppm; ESMS *m/z*: 240.12 ([*M*−Cl]^+^; C_12_H_18_NO_4_ requires 240.12); 262.10 ([*M*−HCl+Na]^+^; C_12_H_17_NaNO_4_ requires 262.11); HRMS *m/z*: 240.1236 ([*M*−Cl]^+^; C_12_H_18_NO_4_ requires 240.1230).

**Compound 11**: 3,5-Pyridinedicarboxylic acid (0.197 g, 1.18 mmol) was suspended in dry CH_2_Cl_2_ (30 mL). Oxalyl chloride (0.75 g, 0.50 mL, 5.89 mmol) and DMF (1 drop) were added. The mixture was stirred at room temperature under N_2_ for 18 h, by which time it had formed a homogenous solution. The solvent was removed on a rotary evaporator and the residue dried under high vacuum for 60 min to afford 3, 5-bis(chlorocarbonyl) pyridine **10** as a waxy, off-white solid, which was redissolved in dry CH_2_Cl_2_ (20 mL). The solution was cooled to 0 °C in an ice bath and a solution of **9**⋅HCl in dry CH_2_Cl_2_ (20 mL) and dry Et_3_N (2.5 mL) was added dropwise by syringe. After addition was complete, the reaction mixture was stirred at 0 °C under N_2_ for 15 min. The ice bath was then removed and the reaction mixture was allowed to stir at room temperature for 18 h. The solution was washed with 10 % citric acid_(aq)_ (2×50 mL), followed by saturated NaHCO_3(aq)_ (2×50 mL) and H_2_O (50 mL). The organic layer was dried over MgSO_4_ and concentrated under reduced pressure. Purification of the residue by column chromatography (2–4 % MeOH in CH_2_Cl_2_ afforded the product as a white solid (0.52 g, 73 %). ^**1**^H NMR (500 MHz; CDCl_3_): *δ*=9.14 (d, ^4^*J*=2.2 Hz, 2 H, py-Ar–H), 8.48 (t, ^4^*J*=2.2 Hz, 1 H, py-Ar–H), 6.80 (t, ^3^*J*=5.6 Hz, 2 H, amide-NH), 6.87–6.83 (m, 8 H, Ar–H), 4.55 (s, 4 H, OC*H*_2_CO_2_Et), 4.25 (quartet, ^3^*J*=7.1 Hz, 4 H, C*H*_2_CH_3_), 4.10 (t, ^3^*J*=5.1 Hz, 4 H, NHCH_2_C*H*_2_O), 3.88–3.85 (m, 4 H, NHC*H*_2_CH_2_O), 1.26 ppm (t, ^3^*J*=7.1 Hz, 6 H, CH_2_C*H*_3_); ^13^C NMR (75 MHz; CDCl_3_): *δ*=169.2, 165.0, 153.2, 152.4, 150.7, 133.6, 129.7, 115.8, 115.4, 66.9, 66.1, 61.4, 39.8, 14.1 ppm; ESMS *m/z*: 632.21 ([*M*+Na]^+^; C_31_H_35_N_3_NaO_10_^+^ requires 632.22); 1241.47 ([2 *M*+Na]^+^; C_62_H_70_N_6_NaO_20_ requires 1241.45); HRMS *m/z*: 632.2216 ([*M*+Na]^+^; C_31_H_35_N_3_NaO_10_ requires 632.2215).

**Compound 12**: Compound **11** (0.50 g, 0.82 mmol) was dissolved in CH_2_Cl_2_ (40 mL) and MeOH (40 mL). A solution of KOH (0.18 g, 3.28 mmol) in H_2_O (7 mL) was added. The solution was stirred at RT under N_2_ for 60 h, during which time a white precipitate formed. H_2_O (40 mL) was added and the organic solvents were removed on a rotary evaporator. The pH of the remaining aqueous suspension was adjusted to 7 by addition of 10 % citric acid_(aq)_ and the solid was collected by filtration, washed with H_2_O (4×15 mL), MeOH (3×5 mL) and CH_2_Cl_2_ (3×10 mL) and dried under high vacuum to afford the product as a white solid (0.40 g, 88 %). ^1^H NMR (300 MHz; [D_6_]DMSO): *δ*=9.06 (d, ^4^*J*=2.2 Hz, 2 H, py-Ar–H), 8.98 (t, ^3^*J*=5.3 Hz, 2 H, amide-NH), 8.57 (t, ^4^*J*=2.2 Hz, 1 H, py-Ar–H), 6.85 (d, ^3^*J*=9.1 Hz, 4 H, hydroquinone-Ar–H), 6.75 (d, ^3^*J*=9.1 Hz, 4 H, hydroquinone-Ar–H), 4.38 (s, 4 H, OC*H*_2_CO_2_H), 4.07 (t, ^3^*J*=5.3 Hz, 4 H, OC*H*_2_CH_2_NH), 3.64–3.59 ppm (m, 4 H, OCH_2_C*H*_2_NH); ESMS *m/z*: 554.16 (M+H]^+^; C_27_H_28_N_3_O_10_ requires 554.18); 576.15 ([*M*+Na]^+^; C_27_H_27_N_3_NaO_10_ requires 576.16); HRMS *m/z*: 576.1641 ([*M*−H]^−^; C_27_H_26_N_3_O_10_ requires 552.1624).

**3,5-Pyridine bis(amide)-strapped porphyrin macrocycle 13**: Dry, de-gassed DMF was added to a flask containing **Zn**⋅**3 a** (0.14 g, 0.25 mmol), compound **12** (0.14 g, 0.25 mmol), *N*-(3-dimethylaminopropyl)-*N*′-ethylcarbodiimide hydrochloride (0.12 g, 0.63 mmol), 1-hydroxybenzotriazole hydrate (0.077 g, ca. 0.57 mmol) and 4-(dimethylamino)pyridine (0.030 g, 0.25 mmol). The mixture was sonicated briefly and then stirred vigorously at room temperature under N_2_ for 60 h. After removal of the solvent under reduced pressure, the residual solid was dissolved in 9:1 CH_2_Cl_2_/MeOH mixture (100 mL), dry-loaded onto silica and purified by column chromatography (1–5 % MeOH in CH_2_Cl_2_) to afford the product as a purple solid (0.12 g, 44 %). ^1^H NMR (500 MHz; [D_6_]DMSO): *δ*=10.43 (s, 2 H, porphyrin-*meso*-H), 9.51 (d, ^3^*J*=4.4 Hz, 4 H, porphyrin-β-pyrrole-H), 8.83 (d, ^3^*J*=4.4 Hz, 4 H, porphyrin-β-pyrrole-H), 8.70 (s, 2 H, amide-NH), 8.60 (d, ^3^*J*=8.3 Hz, 2 H, Ar–H), 8.46 (s, 2 H, py-Ar–H), 8.43 (br t, 2 H, amide-NH), 8.15 (s, 1 H, py-Ar–H), 8.04 (dd, ^3^*J*=7.3 Hz, ^4^*J*=1.5 Hz, 2 H, Ar–H), 7.93–7.89 (m, 2 H, Ar–H), 7.65–7.61 (m, 2 H, Ar–H), 6.21 (d, ^3^*J*=9.0 Hz, 4 H, hydroquinone-Ar–H), 5.28 (d, ^3^*J*=9.0 Hz, 4 H, hydroquinone-Ar–H), 3.92 (t, ^3^*J*=4.9 Hz, 4 H, OC*H*_2_CH_2_NH), 3.76 (s, 4 H, OC*H*_2_CONH), 3.56–3.53 ppm (m, 4 H, OCH_2_C*H*_2_NH); ^13^C NMR (75 MHz, [D_6_]DMSO): *δ*=166.5, 164.6, 152.7, 150.2, 150.1, 149.4, 149.3, 137.6, 135.3, 133.2, 133.1, 132.8, 131.0, 129.1, 128.9, 123.1, 121.1, 114.9, 114.5, 113.2, 106.3, 66.7, 66.6 ppm, one ^13^C signal is coincident with [D_6_]DMSO; ESMS *m/z*: 1094.29 ([*M*+Na]^+^; C_59_H_45_N_9_NaO_8_Zn requires 1094.26); HRMS *m/z*: 1094.2452 ([*M*+Na]^+^; C_59_H_45_N_9_NaO_8_Zn requires 1094.2575). Single crystals suitable for X-ray structural determination were grown by layered diffusion of hexane into a CH_2_Cl_2_/MeOH solution of the macrocycle. Single crystal data: C_59_H_45_N_9_O_8_Zn⋅CH_2_Cl_2_, *M*r=1158.37; triclinic, *P*$\bar 1$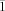; *a*=14.0873(8), *b*=14.2958(7), *c*=15.5922(9) Å; *α*=63.513(5), *β*=71.193(5), *γ*=82.978(4)°; *V*=2659.6(3) Å^3^; data/restraints/parameters: 7853/0/721; *R*_int_=0.085; final *R*_1_=0.057 (*I*>2*σ*(*I*)); *wR*_2_=0.147 (*I*>2*σ*(*I*)); Δρ_min_,_max_=−0.77, +0.86 e Å^−3^.

**3,5-Pyridinium bis(amide)-strapped porphyrin macrocycle 14⋅Cl**: Macrocycle **13** (0.105 g, 0.098 mmol) was dissolved in dry, de-gassed DMF (6 mL). NaHCO_3_ (0.058 g, 0.68 mmol) was added, followed by MeI (0.34 g, 0.15 mL, 2.41 mmol). The reaction mixture was heated at 80 °C under N_2_ for 2 h, using a reflux condenser to prevent loss of MeI. After cooling to room temperature, the excess MeI was removed on a rotary evaporator. The remaining DMF solution was diluted with CH_2_Cl_2_ (100 mL) and then washed with 1 M NH_4_Cl_(aq)_ (6×75 mL) followed by H_2_O (2×75 mL). After concentration of the organic layer under reduced pressure, and recrystallisation of the residual solid (CH_2_Cl_2_/MeOH/hexane), the product was obtained as a purple solid (0.11 g, 99 %). ^1^H NMR (500 MHz, [D_6_]DMSO): *δ*=10.42 (s, 2 H, porphyrin-*meso*-H), 9.48 (d, ^3^*J*=4.4 Hz, 4 H, porphyrin-β*-*pyrrole-H), 9.40 (s, 2 H, pyridinium-Ar–H), 9.14 (br s, 2 H, amide-NH), 8.99 (s, 1 H, pyridinium-Ar–H), 8.75 (d, ^3^*J*=4.4 Hz, 4 H, porphyrin-β*-*pyrrole-H), 8.57 (d, ^3^*J*=9.2 Hz, 2 H, porphyrin-*meso*-Ar–H), 8.34 (s, 2 H, amide-NH), 8.03 (d, ^3^*J*=7.6 Hz, 2 H, porphyrin-*meso*-Ar–H), 7.90–7.86 (m, 2 H, porphyrin-*meso*-Ar–H), 7.62–7.58 (m, 2 H, porphyrin-*meso*-Ar–H), 6.30 (d, ^3^*J*=9.0 Hz, 4 H, hydroquinone-Ar–H), 5.29 (d, ^3^*J*=9.0 Hz, 4 H, hydroquinone-Ar–H), 4.30 (s, 3 H, methylpyridinium-CH_3_), 3.98 (t, ^3^*J*=4.8 Hz, 4 H, CH_2_), 3.80 (s, 4 H, CH_2_), 3.69–3.66 ppm (m, 4 H, CH_2_); ^13^C NMR (125 MHz; [D_6_]DMSO): *δ*=166.5, 161.3, 152.6, 150.1, 149.4, 149.2, 147.1, 140.3, 137.6, 135.2, 133.2, 132.8, 132.7, 131.0, 128.9, 123.1, 121.0, 114.9, 114.4, 113.1, 106.3, 66.6, 66.4, 48.4 ppm, one ^13^C signal is coincident with [D_6_]DMSO; ESMS *m/z*: 1086.32 ([*M*−Cl]^+^; C_60_H_48_N_9_O_8_Zn requires 1086.29); HRMS *m/z*: 1086.2929 ([*M*−Cl]^+^; C_60_H_48_N_9_O_8_Zn requires 1086.2912). Single crystals of the pyridine solvate suitable for X-ray structural determination were grown by layered diffusion of hexane into a CH_2_Cl_2_/MeOH/pyridine solution of the macrocycle. Single crystal data: C_65_H_53_N_10_O_8_Zn⋅Cl, *M*r=1203.03; monoclinic, *P*2_1_/*c*; *a*=10.1084(3), *b*=21.8767(7), *c*=31.8505(12) Å; *α*=*γ*=90°, *β*=90,349(3)°; *V*=7043.2(4) Å^3^; data/restraints/parameters: 11 350/0/766; *R*_int_=0.100; final *R*_1_=0.077 (*I*>2*σ*(*I*)); *wR*_2_=0.182 (*I*>2*σ*(*I*)); Δρ_min_,_max_=−0.61, +0.51 e Å^−3^.

**Chloride catenane 16⋅Cl**: 3,5-Pyridinedicarboxylic acid (0.014 g, 0.081 mmol) was suspended in dry CH_2_Cl_2_ (3 mL). Oxalyl chloride (0.051 g, 0.034 mL, 0.41 mmol) and DMF (1 drop) were added and the mixture was stirred at room temperature under N_2_ until it had formed a homogenous solution (2.5 h). After removal of the solvent on a rotary evaporator, the residual off-white solid was dried under high vacuum for 3 h and then re-dissolved in dry CH_2_Cl_2_ (2.5 mL). Et_3_N (0.041 g, 0.057 mL, 0.41 mmol) was added and the solution was added dropwise to a solution of compound **15** (0.038 g, 0.081 mmol) and macrocycle **14**⋅Cl (0.040 g, 0.032 mmol) in dry CH_2_Cl_2_ (10 mL). The reaction mixture was stirred at room temperature under N_2_ for 90 min, then diluted with CH_2_Cl_2_ (5 mL). The solution was washed sequentially with H_2_O (10 mL), saturated NaHCO_3(aq)_ (10 mL) and brine (10 mL), dried over MgSO_4_ and concentrated on a rotary evaporator. The residue was purified by preparative thin layer chromatography (SiO_2_; 8 % MeOH in CH_2_Cl_2_, then 5 % MeOH in EtOAc) and recrystallisation (CH_2_Cl_2_/MeOH, then CH_2_Cl_2_/MeOH/hexane) to give the product as a purple solid (0.017 g, 30 %). ^1^H NMR (500 MHz; [D_6_]DMSO): *δ*=10.34 (s, 2 H, porphyrin-*meso*-H), 9.52 (d, ^3^*J*=4.6 Hz, 4 H, porphyrin-ß-pyrrole-H), 9.04 (s, 1 H, pyridinium-Ar–H), 8.97 (s, 2 H, pyridinium-Ar–H), 8.96 (s, 2 H, amide-NH), 8.91 (d, 4 H, ^3^*J*=4.6 Hz, porphyrin-ß-pyrrole-H), 8.52 (d, ^3^*J*=8.0 Hz, 2 H, Ar–H), 8.31 (br s, 2 H, amide-NH), 8.28 (s, 1 H, py-Ar–H), 7.91 (t, ^3^*J*=8.0 Hz, 2 H, Ar–H), 7.86 (d, ^3^*J*=7.4 Hz, 2 H, Ar–H), 7.72 (br s, 2 H, amide-NH), 7.59 (t, ^3^*J*=7.4 Hz, 2 H, Ar–H), 6.28 (d, ^3^*J*=9.0 Hz, 4 H, hydroquinone-Ar–H), 6.14 (d, ^3^*J*=8.2 Hz, 4 H, hydroquinone-Ar–H), 6.03 (d, ^3^*J*=9.0 Hz, 4 H, hydroquinone-Ar–H), 5.45 (d, ^3^*J*=8.2 Hz, 4 H, hydroquinone-Ar–H), 4.47 (s, 3 H, methylpyridinium-CH_3_), 3.76 (br t, 4 H, CH_2_), 3.72 (s, 4 H, CH_2_), 3.56 (br t, 4 H, CH_2_), 3.50–3.46 (m, 16 H, CH_2_), 3.44–3.42 (m, 4 H, CH_2_), 3.02–2.99 ppm (m, 4 H, CH_2_), signal corresponding to the external pyridinium proton 16 is not observed in [D_6_]DMSO owing to signal broadening; ^13^C NMR (125 MHz; [D_6_]DMSO): *δ*=166.4, 162.4, 159.9, 152.3, 152.2, 151.5, 150.3, 149.4, 149.2, 147.2, 145.8, 137.2, 136.0, 134.2, 132.8, 132.1, 131.7, 131.2, 128.7, 126.7, 123.6, 122.9, 114.6, 114.4, 114.3, 114.0, 113.8, 106.2, 69.9, 69.8, 69.0, 67.3, 65.0, 48.6 ppm, one ^13^C signal is coincident with [D_6_]DMSO; ESMS *m/z*: 1681.49 ([*M*−Cl]^+^; C_91_H_85_N_12_O_17_Zn requires 1681.54); HRMS *m/z*: 1683.5339 ([*M*−Cl]^+^; C_91_H_85_N_12_O_17_Zn requires 1683.5419). Single crystals suitable for X-ray structural determination were grown by layered diffusion of hexane into a CH_2_Cl_2_/MeOH solution of the catenane. Single crystal data: C_91_H_85_N_12_O_17_Zn⋅2(CH_2_Cl_2_)⋅Cl, *M*r=1889.44; triclinic, *P*$\bar 1$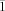; *a*=14.5199(2), *b*=16.4604(2), *c*=21.6127(4) Å; *α*=99.0338(12), *β*=104.2010(14), *γ*=92.0154(11)°; *V*=4930.86(13) Å^3^; data/restraints/parameters: 15 424/0/1153; *R*_int_=0.025; final *R*_1_=0.037 (*I*>2*σ*(*I*)); *wR*_2_=0.087 (*I*>2*σ*(*I*)); Δρ_min_,_max_=−0.69, +0.90 e Å^−3^.

**Hexafluorophosphate catenane 16⋅PF_6_**: The chloride catenane **16**⋅Cl (0.009 g, 0.005 mmol) was dissolved in DMSO (4 mL). The flask was wrapped with foil to protect the contents from light and a solution of AgNO_3_ (0.004 g, 0.026 mmol) in H_2_O (0.16 mL) was added. After stirring at room temperature under N_2_ for 45 min, the solution was filtered through a plug of celite and then added dropwise to 0.05 M NH_4_PF_6(aq)_ (60 mL), which resulted in the formation of a purple precipitate. The precipitate was collected by filtration, washed with H_2_O (6×7.5 mL), EtOH (3×2 mL) and hexane (3×5 mL) and dried under vacuum to yield the product as a purple solid (0.007 g, 73 %). ^1^H NMR (500 MHz, [D_6_]DMSO): *δ*=10.33 (s, 2 H, porphyrin-*meso*-H), 9.49 (d, ^3^*J*=4.2 Hz, 4 H, porphyrin-β*-*pyrrole-H), 9.08 (s, 2 H, pyridinium-Ar–H), 8.84–8.81 (m, 5 H, 4 porphyrin-β*-*pyrrole-H*+*1 pyridinium-Ar–H), 8.72 (s, 2 H, amide-NH), 8.63 (br s, 2 H, amide-NH), 8.49 (br s, 2 H, amide-NH), 8.39 (d, ^3^*J*=8.0 Hz, 2 H, porphyrin-*meso*-Ar–H), 8.32 (s, 1 H, py-Ar–H), 7.93 (d, ^3^*J*=6.9 Hz, 2 H, porphyrin-*meso*-Ar–H), 7.88–7.85 (m, 2 H, porphyrin-*meso*-Ar–H), 7.61–7.58 (m, 2 H, porphyrin-*meso*-Ar–H), 6.39 (d, ^3^*J*=7.8 Hz, 4 H, hydroquinone-Ar–H), 6.28 (d, ^3^*J*=8.0 Hz, 4 H, hydroquinone-Ar–H), 6.00 (d, ^3^*J*=8.0 Hz, 4 H, hydroquinone-Ar–H), 5.87 (d, ^3^*J*=7.8 Hz, 4 H, hydroquinone-Ar–H), 4.23 (s, 3 H, methylpyridinium-CH_3_), 3.83–3.81 (m, 4 H, CH_2_), 3.71–3.69 (m, 4 H, CH_2_), 3.64 (s, 4 H, CH_2_), 3.55–3.51 (m, 4 H, CH_2_), 3.42–3.39 (m, 4 H, CH_2_), 3.08–3.02 (m, 8 H, CH_2_), 2.99–2.97 3.83–3.81 ppm (m, 4 H, CH_2_); ^19^F NMR (470 MHz, [D_6_]DMSO): *δ*=−70.1 ppm (d, ^2^*J*(F,P)=711 Hz), ^31^P NMR (203 MHz, [D_6_]DMSO): *δ*=−144.2 ppm (septet, ^2^*J*(F,P)=711 Hz); ESMS *m/z*: 1683.51 ([*M*−PF_6_]^+^; C_91_H_85_N_12_O_17_Zn requires 1683.54). Single crystals suitable for X-ray structural determination were grown by layered diffusion of di-isopropyl ether into an acetone solution of the catenane. Single crystal data: C_91_H_85_N_12_O_17_Zn⋅0.5(PF_6_), *M*r=1756.59; tetragonal, *P*4/*ncc*; *a*=41.5009(6), *b*=41.5009(6), *c*=24.4007(5) Å; *α*=*β*=*γ*=90°, *V*=42 025.9(15) Å^3^; data/restraints/parameters: 10 933/3740/1415; *R*_int_=0.110; final *R*_1_=0.091 (*I*>2*σ*(*I*)); *wR*_2_=0.237 (*I*>2*σ*(*I*)); Δρ_min_,_max_=−0.51, +0.87 e Å^−3^.

## Supporting Information

As a service to our authors and readers, this journal provides supporting information supplied by the authors. Such materials are peer reviewed and may be re-organized for online delivery, but are not copy-edited or typeset. Technical support issues arising from supporting information (other than missing files) should be addressed to the authors.

miscellaneous_information
