# Supplementary material for: Chloride-Anion-Templated Synthesis of a Strapped-Porphyrin-Containing Catenane Host System
Source: Chemistry. 2015 Oct 28;21(49):17664–75. doi: 10.1002/chem.201502721 (PMC4691337; doi:10.1002/chem.201502721)
Supplement: Supplementary file 1 [file chem0021-17664-sd1.pdf]

CHEMISTRY
A European Journal

Supporting Information

# Chloride-Anion-Templated Synthesis of a Strapped-Porphyrin-Containing Catenane Host System

Asha Brown,[a] Matthew J. Langton,[a] Nathan L. Kilah,[b] Amber L. Thompson,[a] and Paul D. Beer*[a]

chem_201502721_sm_miscellaneous_information.pdf

**Contents**

**Synthetic procedures for compounds 2 and 4–7** ---------- **S2**

**NMR spectra of new compounds** ---------- **S4**

Compound **3a** ---------- S4

Compound **3b** ---------- S5

Compound Zn·**3a** ---------- S5

Compound **8** ---------- S6

Compound **9**·Cl ---------- S7

Compound **11** ---------- S8

Compound **12** ---------- S9

Compound **13** ---------- S9

Compound **14**·Cl ---------- S10

Compound **16**·Cl ---------- S11

Compound **16**·$PF_6$ ---------- S14

**Anion recognition studies** ---------- **S16**

$^{1}$H NMR titration experiments ---------- S16

UV-Visible and fluorescence experiments ---------- S16

**References** ---------- **S17**

## Synthesis procedures for compounds 2 and 4–7

### 5,15-Bis-(2-nitrophenyl)porphyrin 2[1]

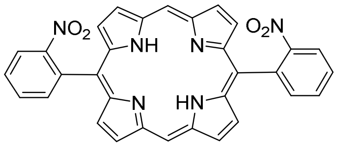


2-Nitrobenzaldehyde (1.55 g, 10.3 mmol) was dissolved in $CH_2Cl_2$ (2 L). EtOH (15 mL) was added and the solution was purged with $N_2$ for 5 minutes, before addition of *meso*-unsubstituted dipyrromethane **1** (1.5 g, 10.3 mmol). After purging with $N_2$ for a further 10 minutes, the flask was wrapped with foil to protect the reaction mixture from light and $BF_3{\cdot}OEt_2$ (0.49 g, 0.42 mL, 3.4 mmol) was added. The solution was then stirred at room temperature for 60 minutes while continuing to bubble $N_2$ through slowly. *p*-Chloranil (5.05 g, 20.5 mmol) was added and the reaction mixture was heated at reflux under $N_2$ for 60 minutes. After cooling to room temperature, $Et_3N$ (10 mL) was added. The reaction mixture was filtered through a plug of silica and the silica plug was washed with $CH_2Cl_2$ until all of the porphyrin product had been eluted. The porphyrin-containing fractions were combined and concentrated on a rotary evaporator and the residue was crystallised twice from $CH_2Cl_2$/MeOH to give the product as a purple solid (0.60 g, 21%). $^1$H NMR (300 MHz; $CDCl_3$): $\delta$=10.32–10.31 (m, 2 H, *meso*-porphyrin-*H*), 9.40–9.38 (m, 4 H, $\beta$-pyrrole-*H*), 8.88–8.86 (m, 4 H, $\beta$-pyrrole-*H*), 8.53–8.49 (m, 2 H, Ar*H*), 8.33–8.29 (m, 2 H, Ar*H*), 8.04–8.01 (m, 4 H, Ar*H*), −3.10 (br s, 2 H, pyrrole-N*H*).

### 3-(4-(Benzyloxy)phenoxy)propanenitrile 4[2]

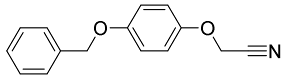


4-(Benzyloxy)phenol (6.00 g, 30.0 mmol) and bromoacetonitrile (3.95 g, 2.30 mL, 33.0 mmol) were dissolved in acetone (100 mL). $K_2CO_3$ (4.56 g, 33.0 mmol) was added. The mixture was heated at reflux under $N_2$ for 18 hours. The reaction mixture was then cooled to r.t. and filtered. The filtrate was concentrated on a rotary evaporator to leave a brown oil, which was purified on a short plug of silica, eluting with $CH_2Cl_2$, to give the product as a pale yellow solid (6.97g, 97%). $^1$H NMR (300 MHz; $CDCl_3$): $\delta$=7.45–7.34 (m, 5 H, benzyl-C*H*), 6.96 (s, 4 H, Ar*H*), 5.05 (s, 2 benzyl- This characterisation data is consistent with that described previously.[3]

### 2-(4-(Benzyloxy)phenoxy)ethanamine 5[2a]

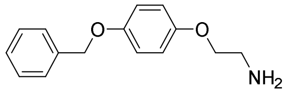


$LiAlH_4$ (1.59 g, 42.0 mmol) was suspended in dry $Et_2O$ (100 mL) and a solution of 3-(4-(benzyloxy)phenoxy)propanenitrile **4** (6.70 g, 28.0 mmol) in $Et_2O$ (100 mL) was added slowly. The mixture was heated at reflux under $N_2$ for 60 minutes. After cooling to r.t., $H_2O$ (1 mL) was added cautiously. 1 M $NaOH_{(aq)}$ (2.5 mL) was then added, followed by $H_2O$ (1.5 mL). The mixture was stirred vigorously at r.t. for 5 minutes and then filtered. The solid was washed with $Et_2O$ (3 x 15 mL). The combined $Et_2O$ filtrate and washings were dried over $MgSO_4$ and concentrated on a rotary evaporator to give the product as an off-white solid (5.73 g, 84%). $^1$H

NMR (300 MHz; $CDCl_3$): $\delta$=7.45–7.33 (m, 5 H, benzyl-C*H*), 6.92 (d, $^3J$ = 9.4 Hz, 2 H, Ar*H*), 6.85 (d, $^3J$ = 9.4 Hz, 2 H, Ar*H*), 5.02 (s, 2 H, benzyl-C$H_2$), 3.95 (t, $^3J$ = 5.1 Hz, 2 H, C$H_2$), 3.07 (t, $^3J$ = 5.1 Hz, 2 H, C$H_2$).

**tert-Butyl (2-(4-(benzyloxy)phenoxy)ethyl)carbamate 6[3]**

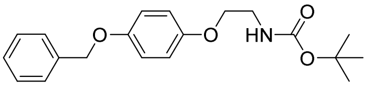

2-(4-(Benzyloxy)phenoxy)ethanamine **5** (2.50 g, 10.3 mmol) was dissolved in dry $CH_2Cl_2$ (250 mL) under $N_2$. Di-*tert*-butyldicarbaonate (2.47 g, 11.3 mmol) was added. The solution was stirred at r.t. under $N_2$ for 18 hours. The solvent was removed on a rotary evaporator. The residual yellow oil was purified on a short plug of silica, eluting with $CH_2Cl_2$, to yield a colourless oil. This was dissolved in boiling hexane (65 mL). The solution was allowed to cool to r.t., before being concentrated to half of its original volume and left to stand at r.t. for 12 hours. The resulting white crystals were collected by filtration, washed with hexane (3 x 10 mL) and dried under vacuum (3.06 g, 87%). $^1$H NMR (300 MHz; $CDCl_3$): $\delta$=7.45–7.33 (m, 5 H, benzyl-Ar*H*), 6.92 (d, $^3J$ = 9.4 Hz, 2 H, Ar*H*), 6.85–6.82 (d, $^3J$ = 9.4 Hz, 2 H, Ar*H*), 5.03 (s, 2 H, benzyl-C$H_2$), 4.99 (br s, 1 H, N*H*), 3.98 (t, $^3J$ = 5.3 Hz, 2 H, C$H_2$), 3.54–3.49 (m, 2 H, C$H_2$), 1.46 (s, 9 H, C$H_3$).

**tert-Butyl (2-(4-hydroxyphenoxy)ethyl)carbamate 7[3]**

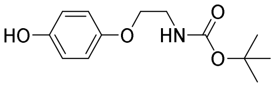

*tert*-Butyl (2-(4-(benzyloxy)phenoxy)ethyl)carbamate **6** (1.50 g, 4.37 mmol) was dissolved in $CHCl_3$ (35 mL). 10% Pd/C (0.150 g, 0.141 mmol of Pd) was added, followed by MeOH (30 mL). The flask was evacuated and back-filled with $H_2$ three times. The reaction mixture was then stirred vigorously at r.t. under an atmosphere of $H_2$ for 18 hours. The solution was filtered through a plug of celite and the filtrate was concentrated to give a pale yellow oil. This was dissolved in $CHCl_3$ (60 mL) and the solution was filtered to remove a small amount of undissolved white solid. The filtrate was concentrated on a rotary evaporator to give a yellow oil, to which was added hexane (30 mL). The mixture was stirred at r.t. for 18 hours. The solid was collected by filtration, washed with hexane (2 x 10 mL) and dried under vacuum to give the product as a white solid (1.05 g, 95%). $^1$H NMR (300 MHz; $CDCl_3$): $\delta$=6.77 (s, 4 H, Ar*H*), 5.01 (br s, 1 H, O*H*), 4.80 (br s, 1 H, N*H*), 3.95 (t, $^3J$ = 5.3 Hz, 2 H, C$H_2$), 3.54–3.48 (m, 2 H, C$H_2$), 1.46 (s, 9 H, C$H_3$); *m/z* (ES): 276.12 ($[M + Na]^+$. $C_{13}H_{19}NNaO_4$ requires 276.12).

## NMR spectra of new compounds

### Compound 3a

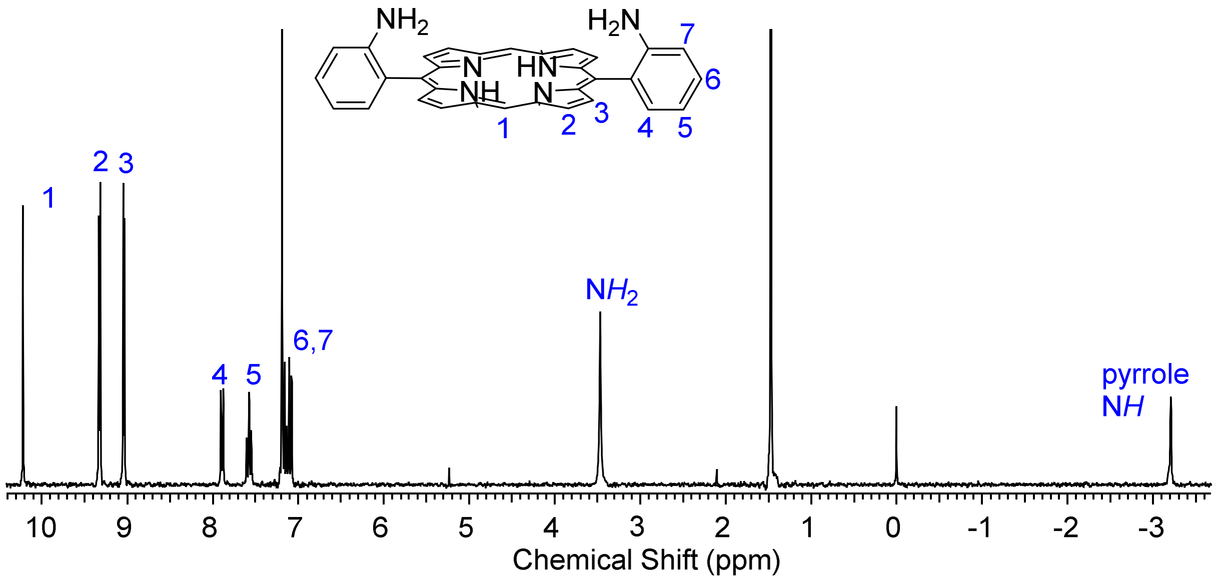


**Fig S1**. $^{1}H$ NMR spectrum of compound **3a** in $CDCl_3$ at 293 K

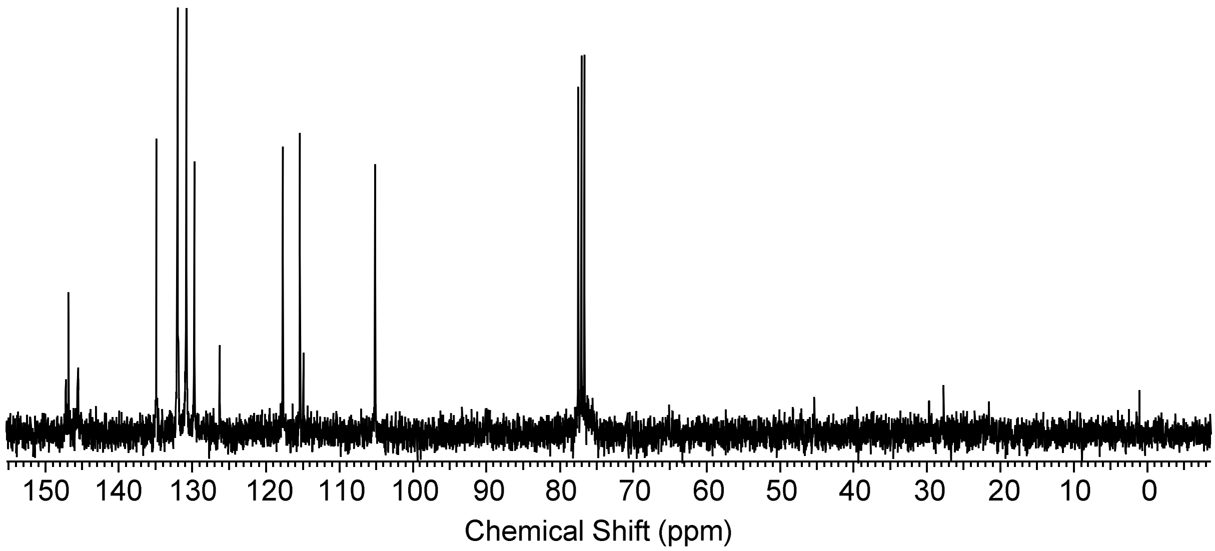


**Fig S2**. $^{13}C$ NMR spectrum of compound **3a** in $CDCl_3$ at 293 K

**Compound 3b**

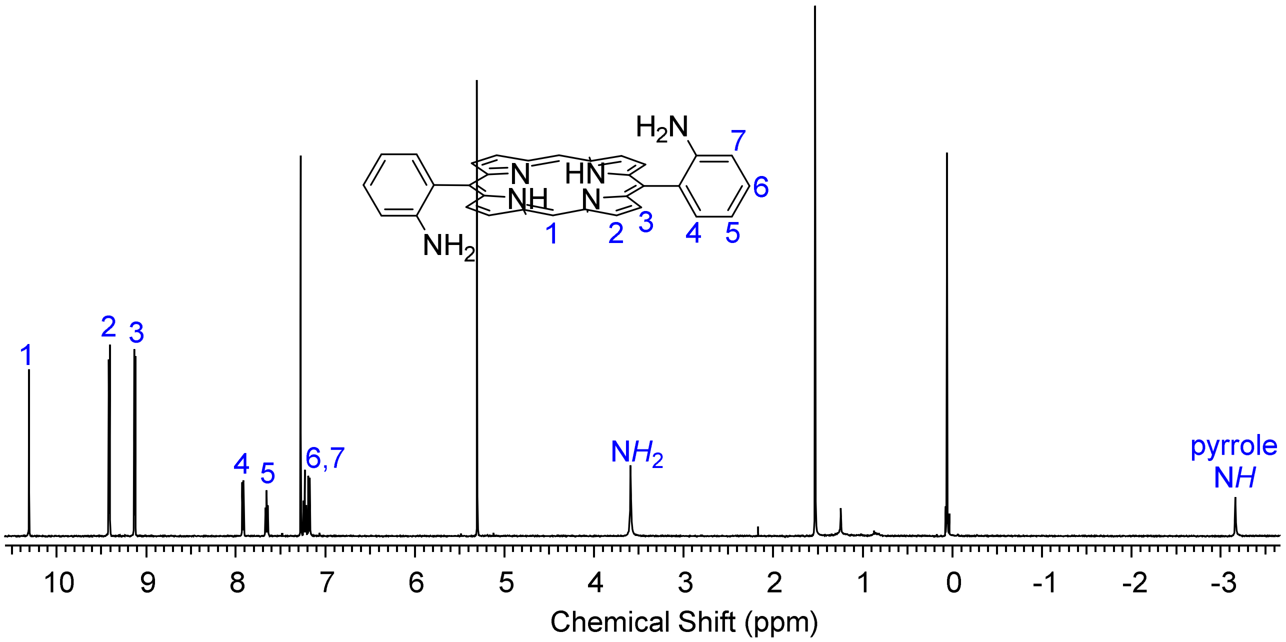


**Fig S3**. $^{1}$H NMR spectrum of compound **3a** in $CDCl_3$ at 293 K

**Compound** Zn·**3a**

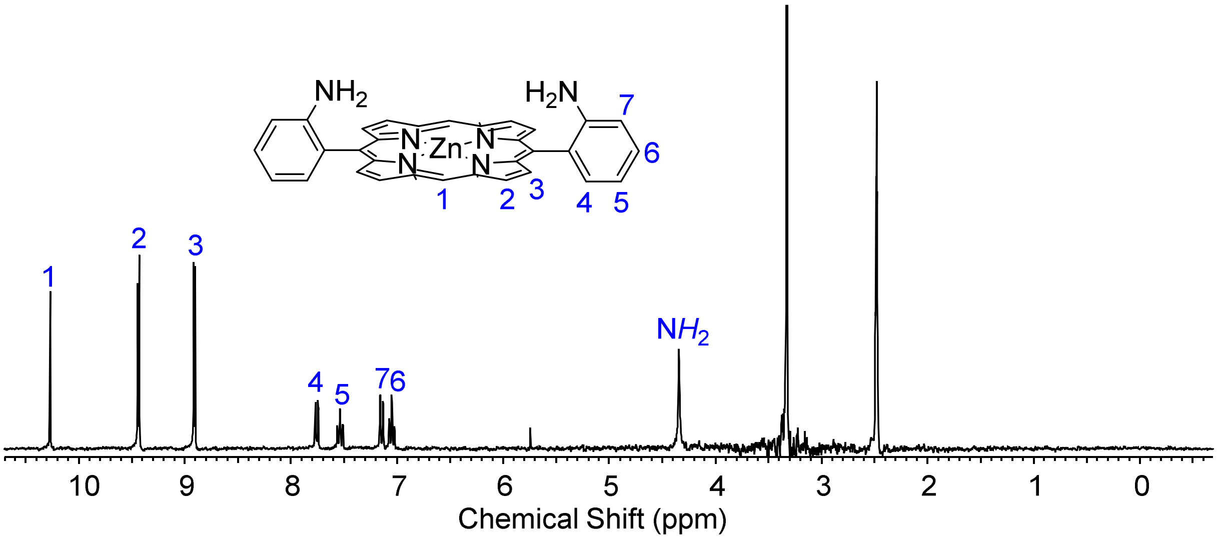


**Fig S4**. $^{1}$H NMR spectrum of compound Zn·**3a** in $[D_6]DMSO$ at 293 K

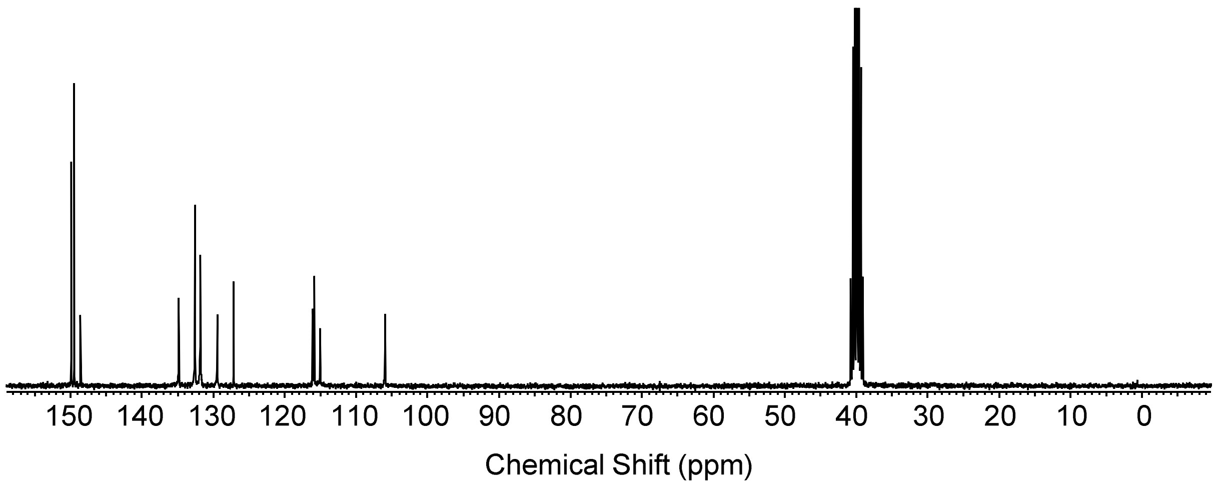


**Fig S5**. $^{13}$C NMR spectrum of compound Zn·**3a** in [D$_6$]DMSO at 293 K

**Compound 8**

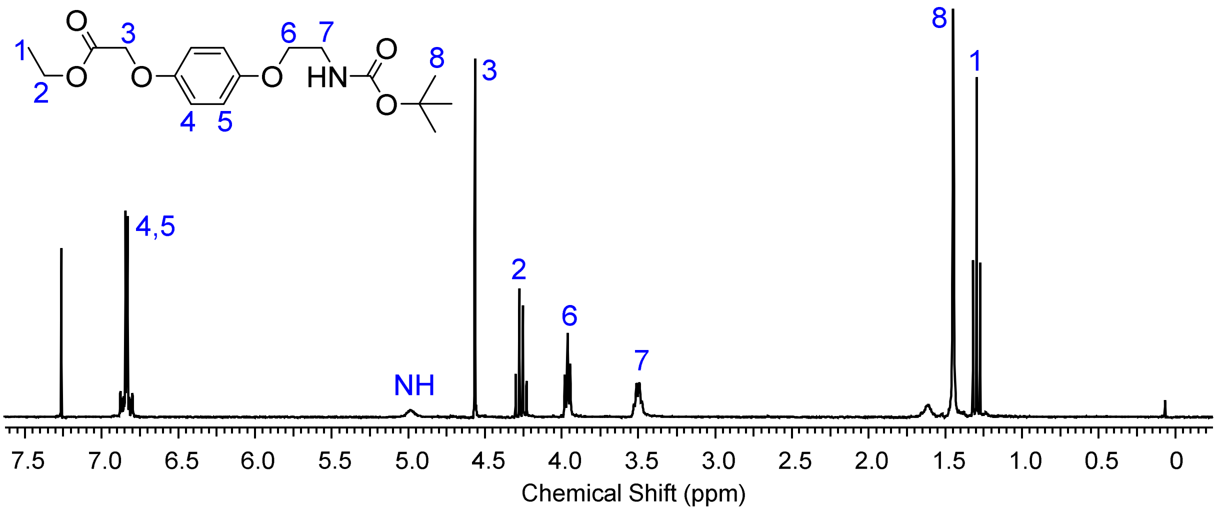


**Fig S6**. $^{1}$H NMR spectrum of compound **8** in $CDCl_3$ at 293 K

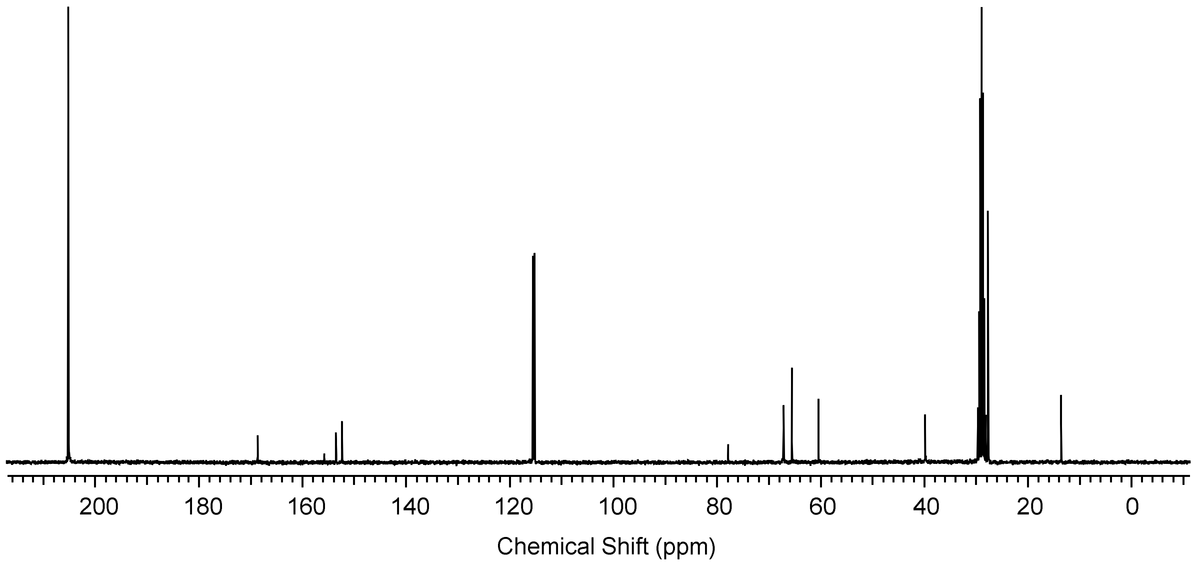


**Fig S7**. $^{13}$C NMR spectrum of compound **8** in [D$_6$]acetone at 293 K

**Compound 9·HCl**

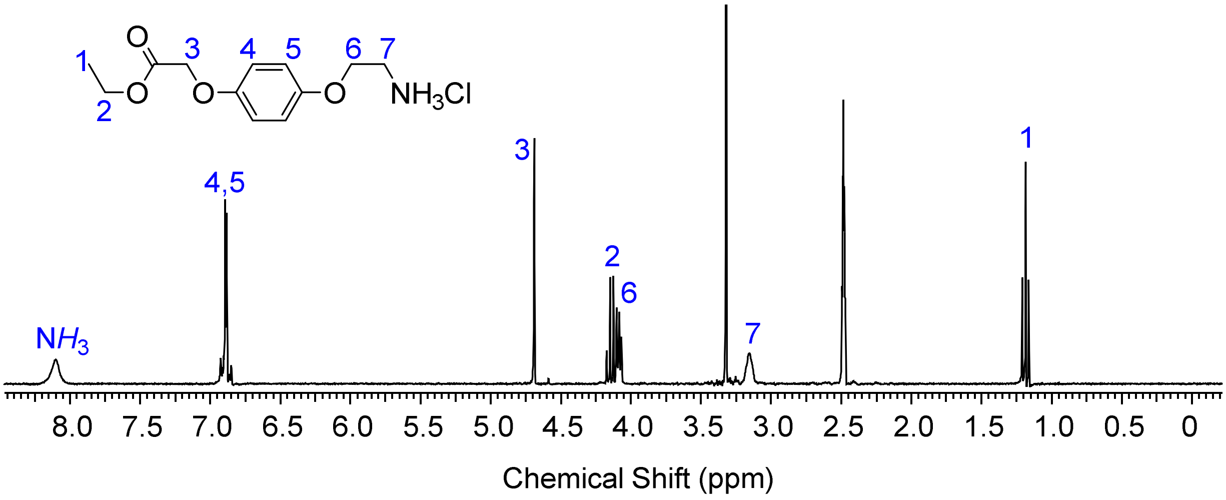


**Fig S8.** ${}^{1}H$ NMR spectrum of compound **9**·HCl in [D6]DMSO at 293 K

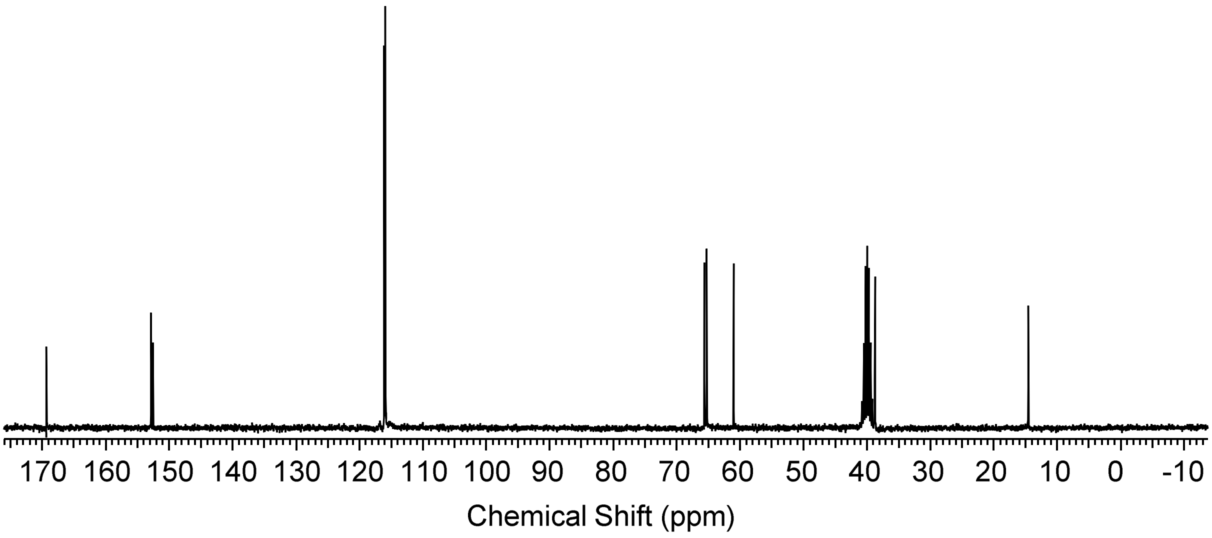


**Fig S9.** ${}^{13}C$ NMR spectrum of compound **9**·HCl in [$D_6$]DMSO at 293 K

**Compound 11**

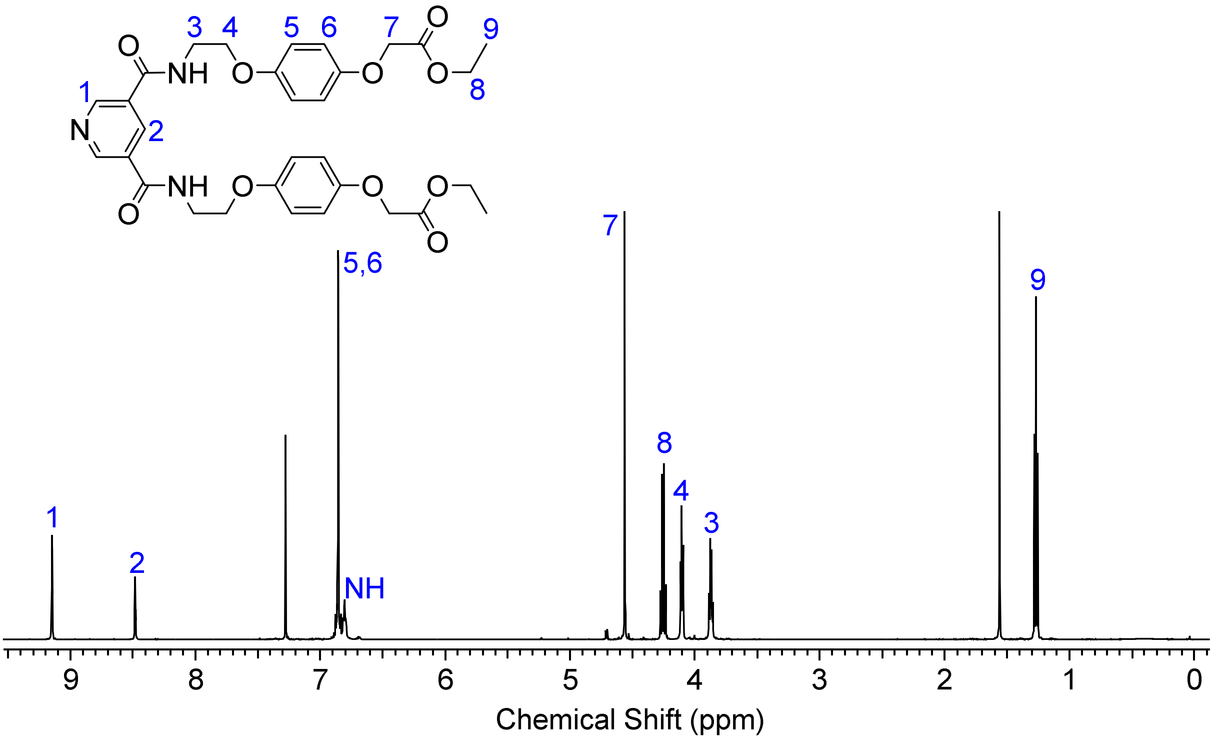


**Fig S10**. $^{1}H$ NMR spectrum of compound **11** in $CDCl_3$ at 293 K

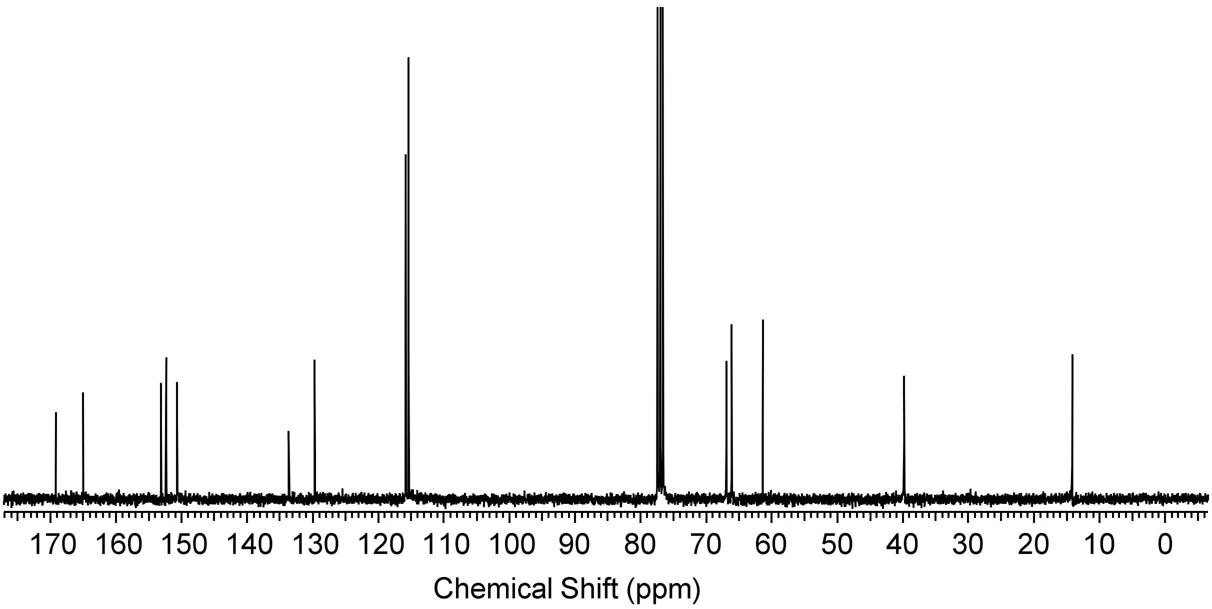


**Fig S11**. $^{13}C$ NMR spectrum of compound **11** in $CDCl_3$ at 293 K

**Compound 12**

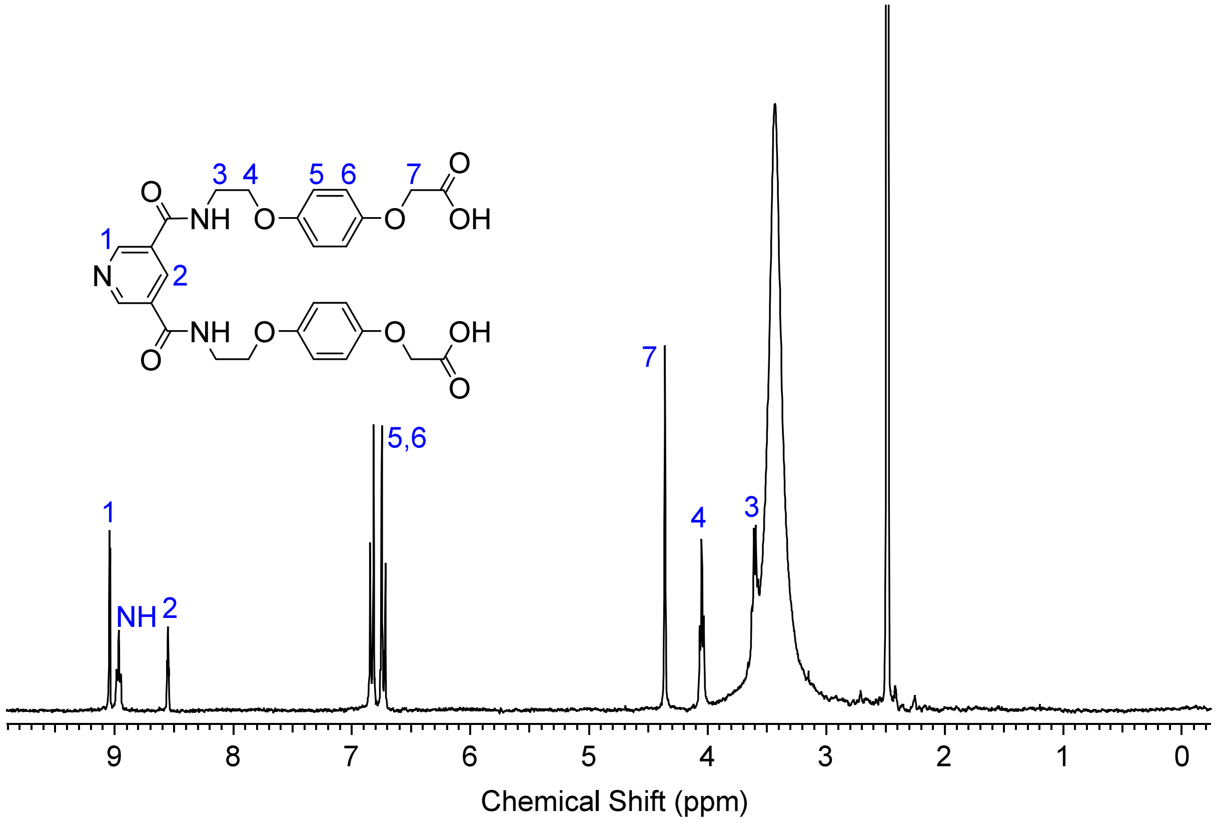


**Fig S12**. $^{1}H$ NMR spectrum of compound **12** in $[D_6]DMSO$ at 293 K

**Compound 13**

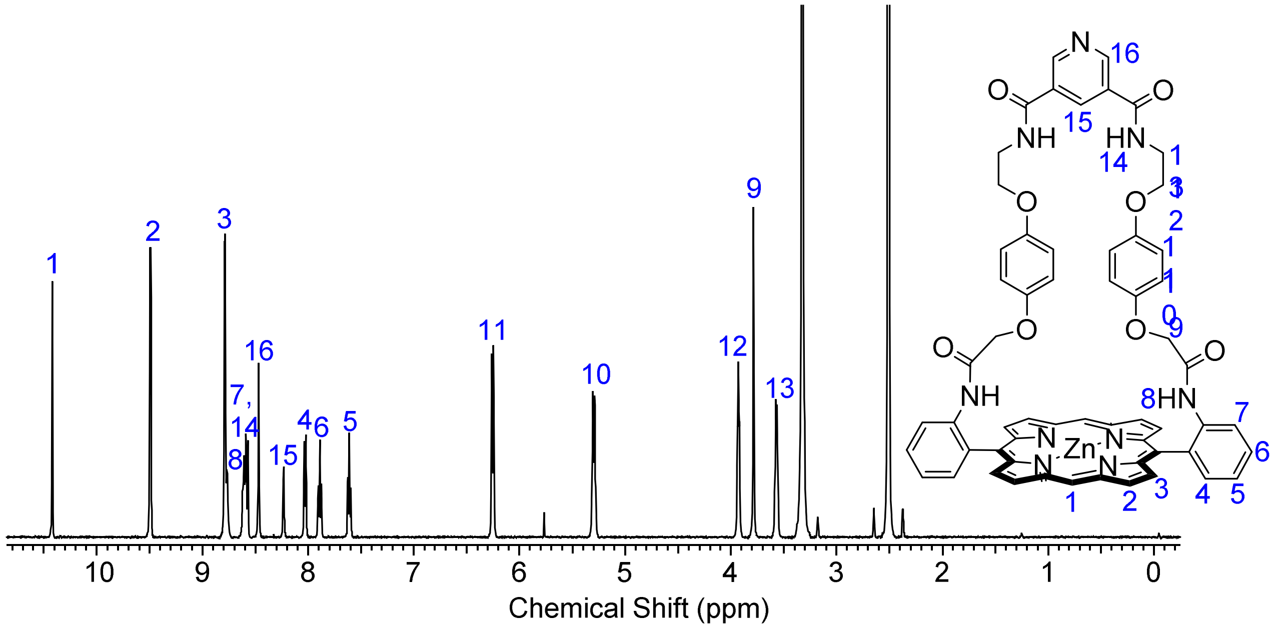


**Fig S13**. $^{1}H$ NMR spectrum of compound **13** in $[D_6]DMSO$ at 293 K

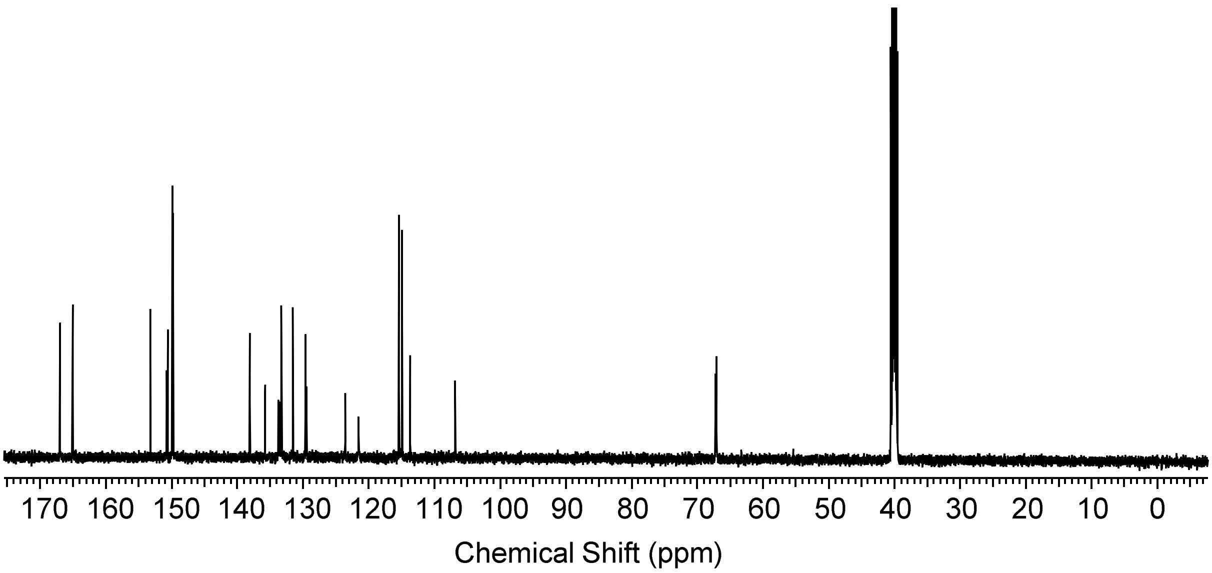


**Fig S14**. $^{1}$13 C NMR spectrum of compound **13** in $[D_6]$DMSO at 293 K

**Compound 14·Cl**

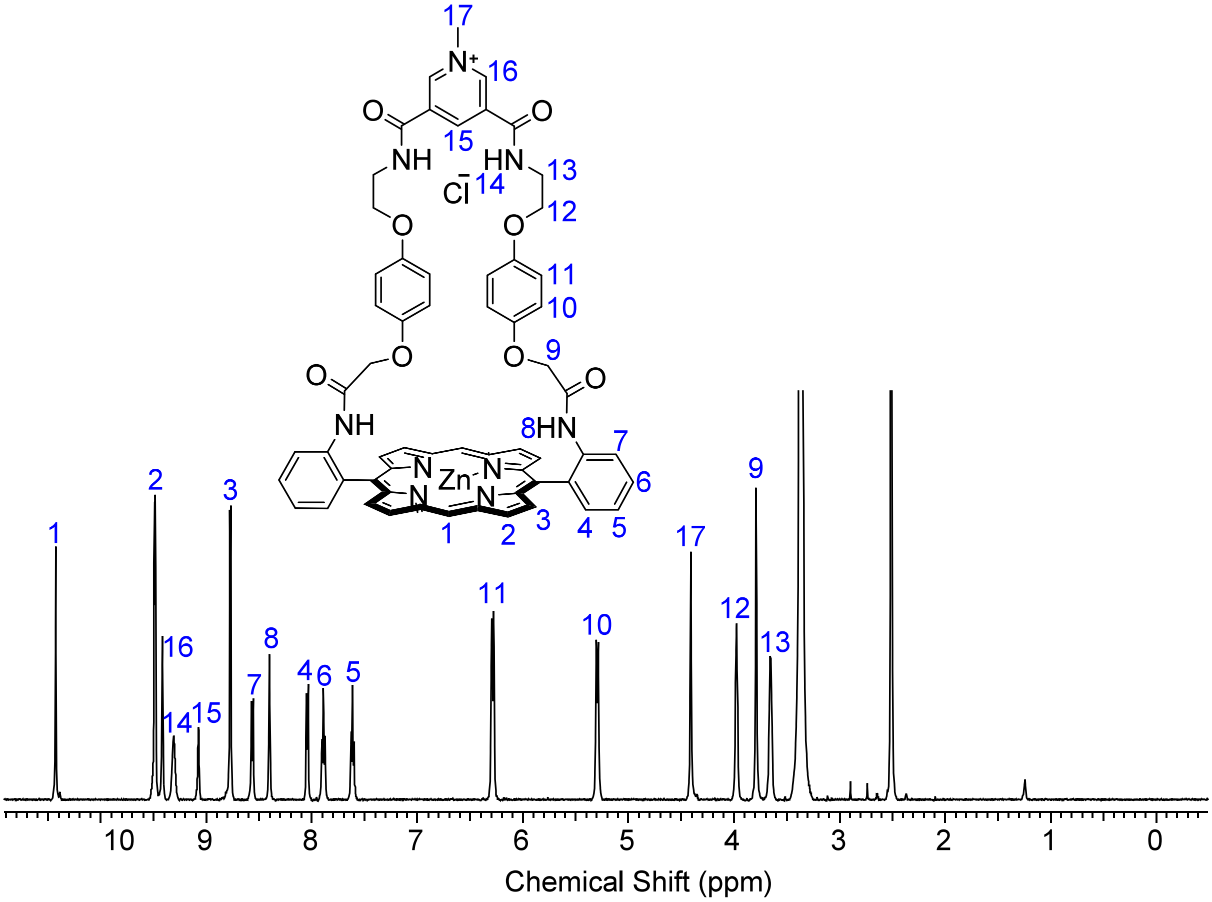


**Fig S15**. $^{1}$H NMR spectrum of compound **14**·Cl in $[D_6]$DMSO at 293 K

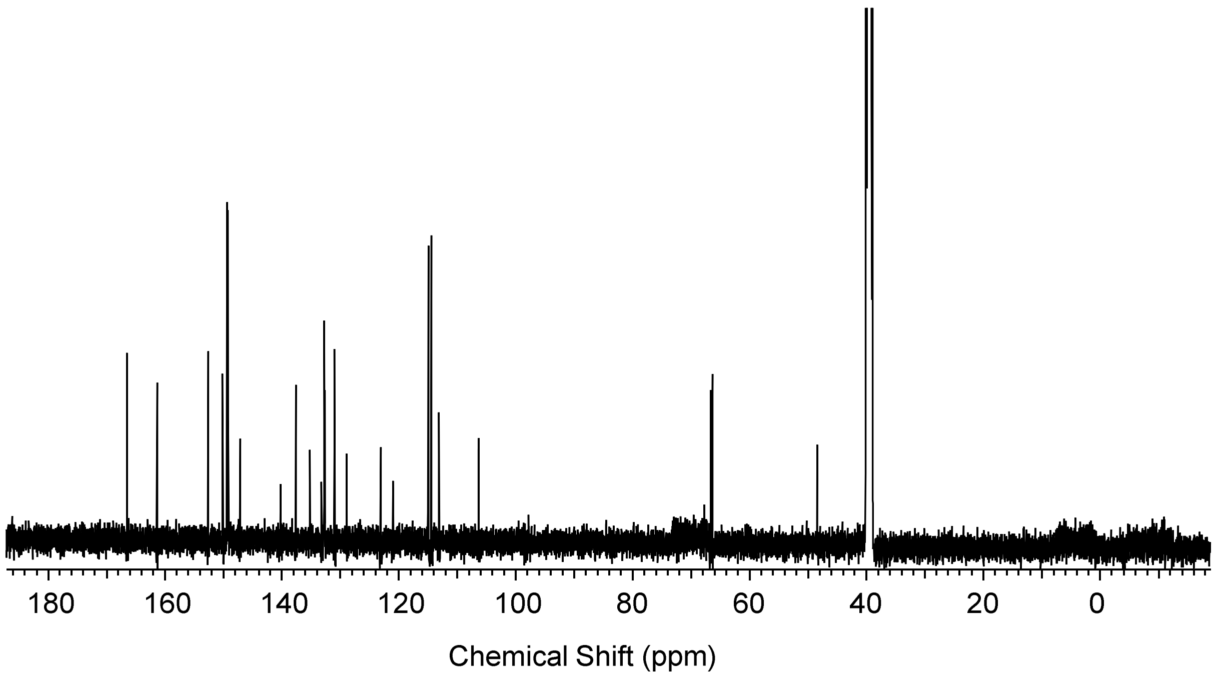


**Fig S16**. $^{13}C$ NMR spectrum of compound **14**·Cl in $[D_6]DMSO$ at 293 K

**Compound 16·Cl**

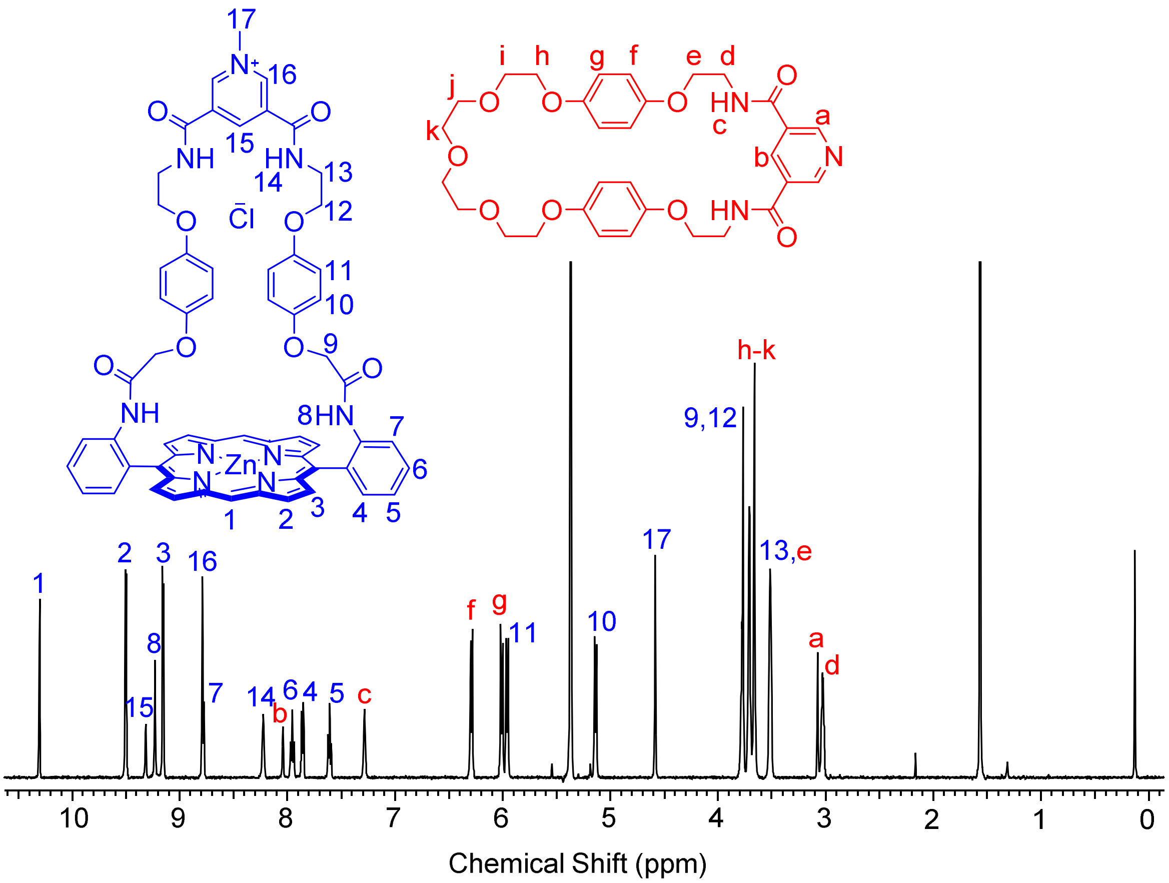


**Fig S17**. $^{1}H$ NMR spectrum of catenane **16**·Cl in $CD_2Cl_2$ at 293 K

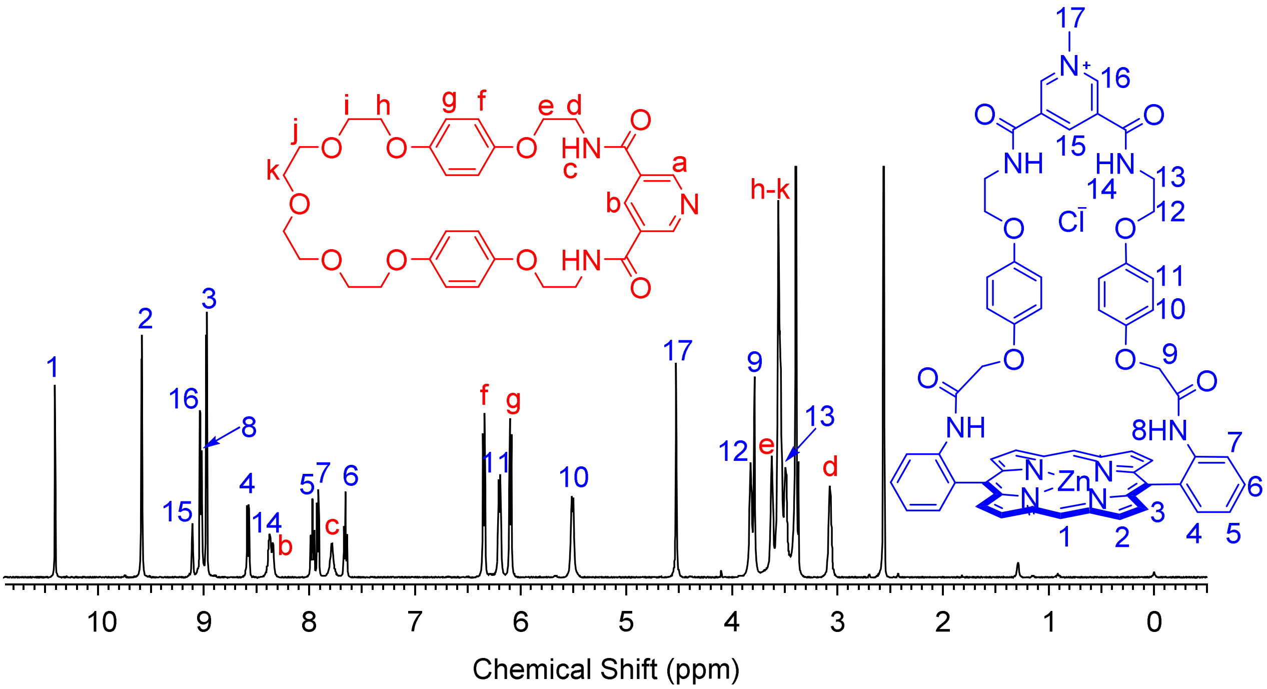


**Fig S18**. $^{1}$H NMR spectrum of catenane **16**·Cl in $[D_6]$DMSO at 293 K

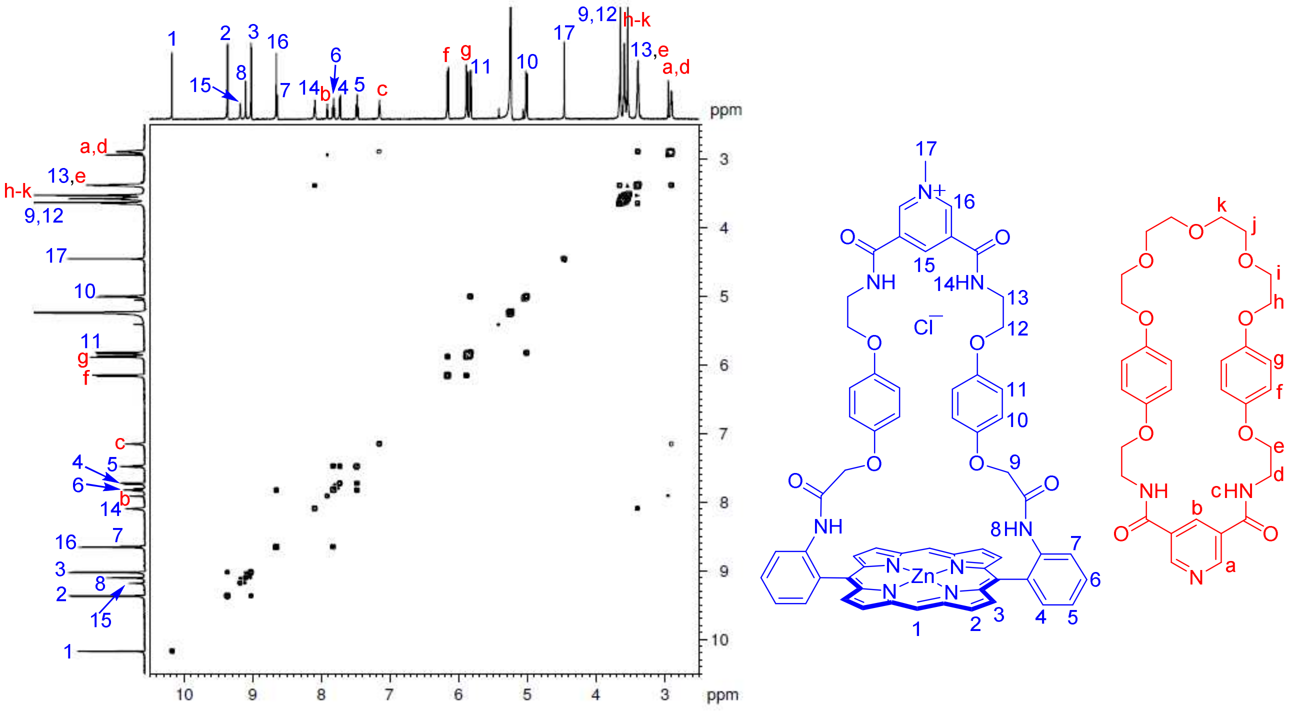


**Fig S19**. $^{1}$H NMR COSY spectrum of catenane **16**·Cl in $CD_2Cl_2$ at 293 K

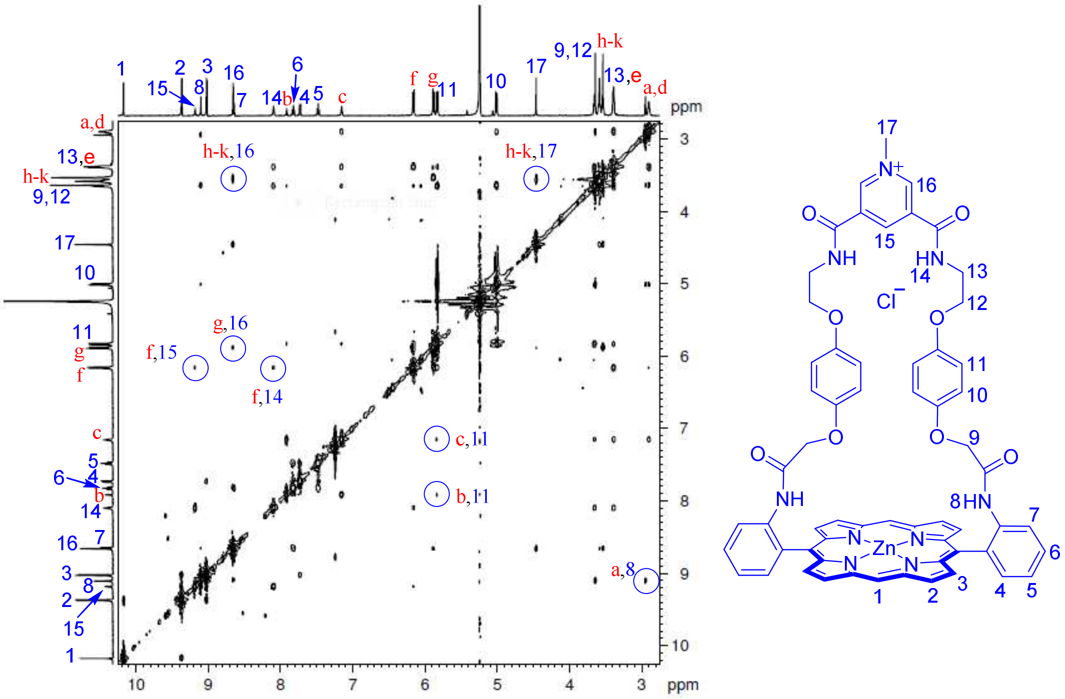


**Fig S20**. $^{1}H$ NMR ROESY spectrum of catenane **16·**Cl in $CD_2Cl_2$ at 293 K. Through-space interactions between the two interlocked macrocyclic components of the catenane are highlighted.

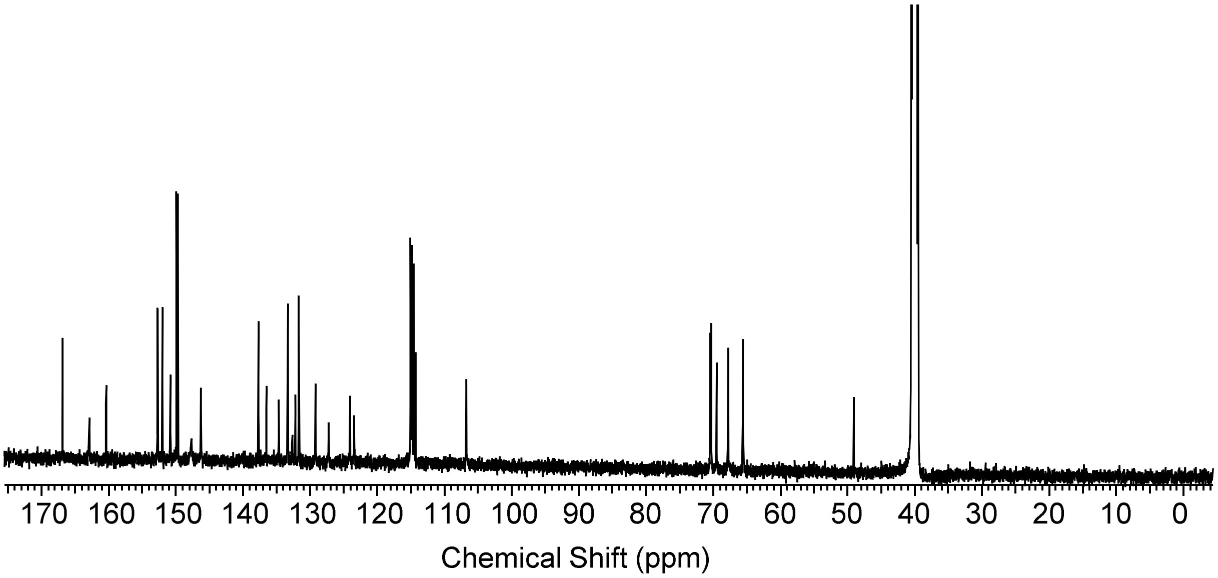


**Fig S21**. $^{13}C$ NMR spectrum of catenane **16·**Cl in [$D_6$]DMSO at 293 K

**Compound 16·$PF_6$**

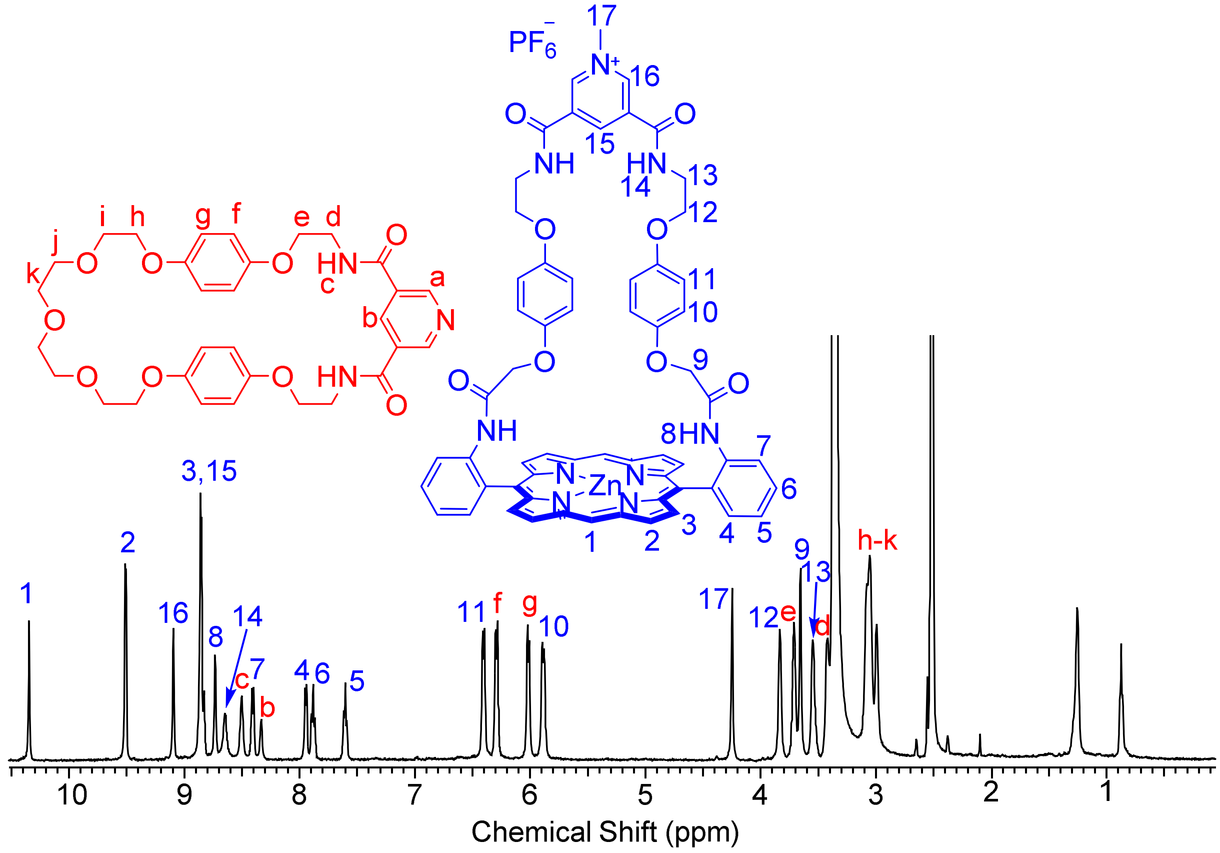


**Fig S22**. $^{1}$H NMR spectrum of catenane **16**·$PF_6$ in [$D_6$]DMSO at 293 K

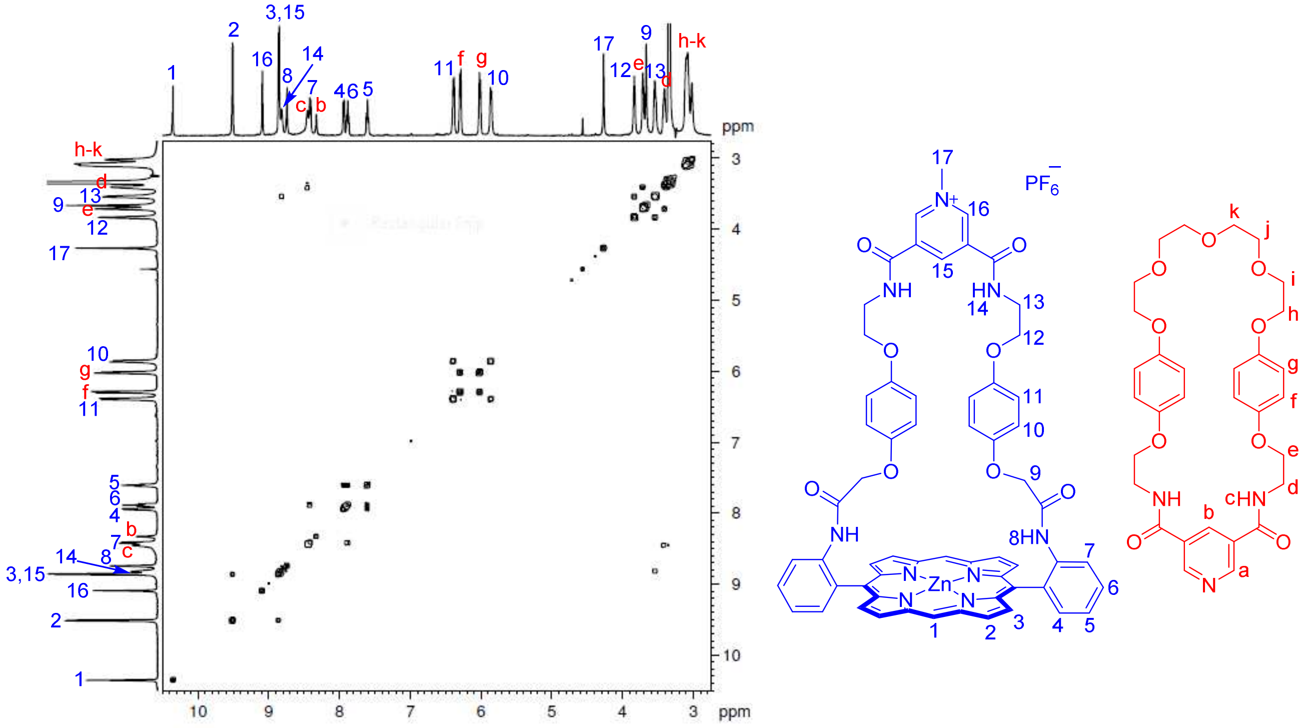


**Fig S23**. $^{1}$H NMR COSYspectrum of catenane **16**·$PF_6$ in [$D_6$]DMSO at 293 K

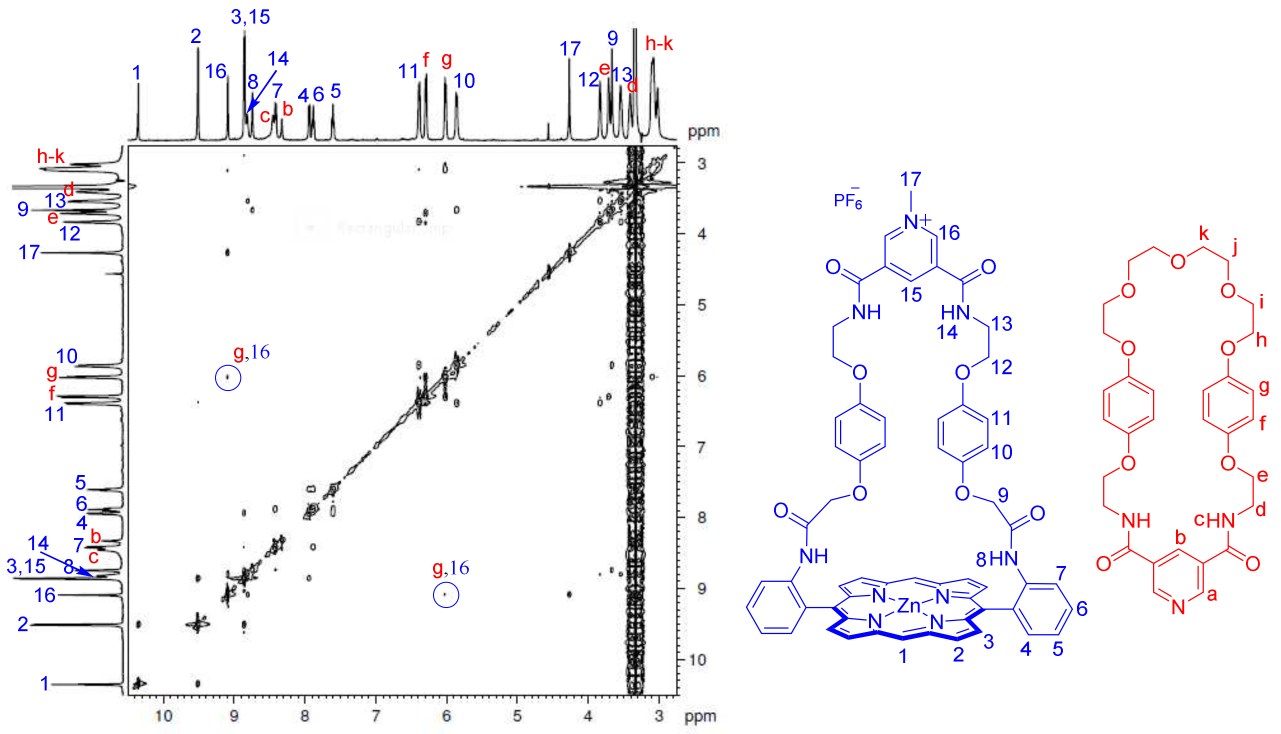

**Fig S24**. $^{1}$H NMR ROESY spectrum of catenane **16**·$PF_6$ in [$D_6$]DMSO at 293 K. A through-space interaction between the pyridinium and hydroquinone groups of the pyridinium-strapped-porphyrin and 3,5-pyridine-bis-amide macrocycle components respectively is highlighted.

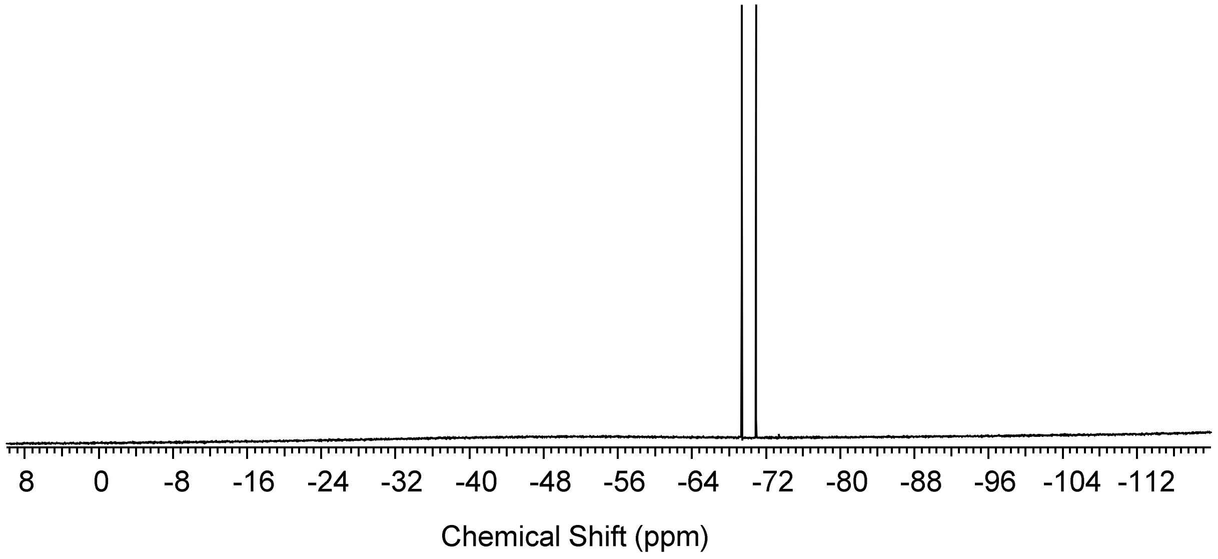


**Fig S25**. $^{19}F$ NMR spectrum of catenane **16**·$PF_6$ in [$D_6$]DMSO at 293 K

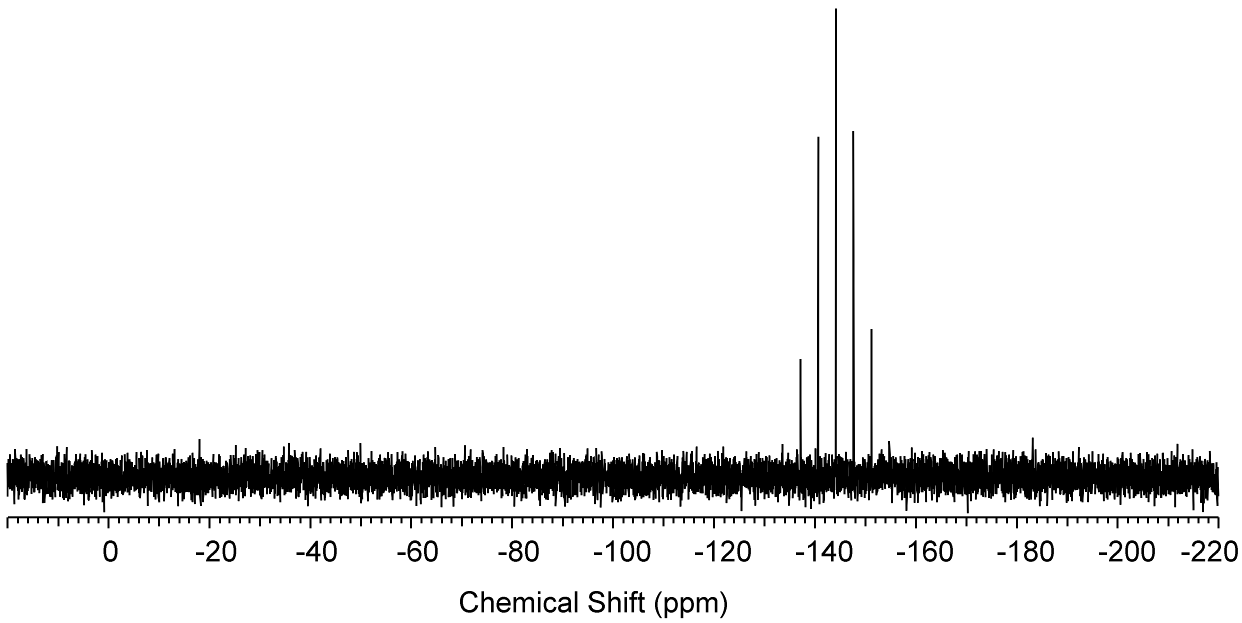


**Fig S26**. $^{31}P$ NMR spectrum of catenane **16**·$PF_6$ in [$D_6$]DMSO at 293 K

## Anion recognition studies

### $^{1}H$ NMR titration experiments

All $^{1}H$ NMR titration experiments were conducted on a Bruker Avance III 500 MHz NMR spectrometer, at 298K. Initial sample volumes were 600 µl. The starting concentration of the host was 1.5 mM. All anions were added as their TBA salts. The concentrations of the TBAX solutions were 45 mM. 17 aliquots of the TBAX solutions were added until a total of 10 equivalents of the anion had been added. Spectra were recorded after each addition, and the sample shaken thoroughly before measurement.

The stability constant for chloride binding was obtained by analysis of the titration data using the WinEQNMR2[4] computer program. Estimates for the binding constant, the limiting chemical shifts and the complex stoichiometry were also added to the input file. The various parameters were refined by non-linear least-squares analysis to achieve the best fit between observed and calculated chemical shifts. The parameters were varied until the values for the stability constants converged. Comparison of the calculated binding isotherm with that obtained experimentally demonstrated that the model used was appropriate.

### UV-Visible and fluorescence experiments

UV-visible titration experiments were conducted using a PG instruments T60U spectrometer. Fluorescence titration experiments were conducted using a Horiba Jobin Yvon FluoroLog3. During each titration experiment, aliquots of a TBAX guest solution were repeatedly added to a solution of the [2]rotaxane host compound in a cuvette. After each addition, the sample was mixed thoroughly and the spectrum was recorded. The concentration of the host compound was kept constant throughout each experiment.

The stability constant for chloride binding was obtained by analysis of the resulting titration data using the SPECFIT[5] computer program. The parameters were refined by global analysis using singular value decomposition and non-linear modelling by the Levenberg-Marquardt method. The parameters were varied until the values for the stability constants converged. Comparison of the theoretical binding isotherms, calculated concentration profiles and the predicted spectrum of the catenane·chloride complex with the experimental data confirmed that the model used was correct.

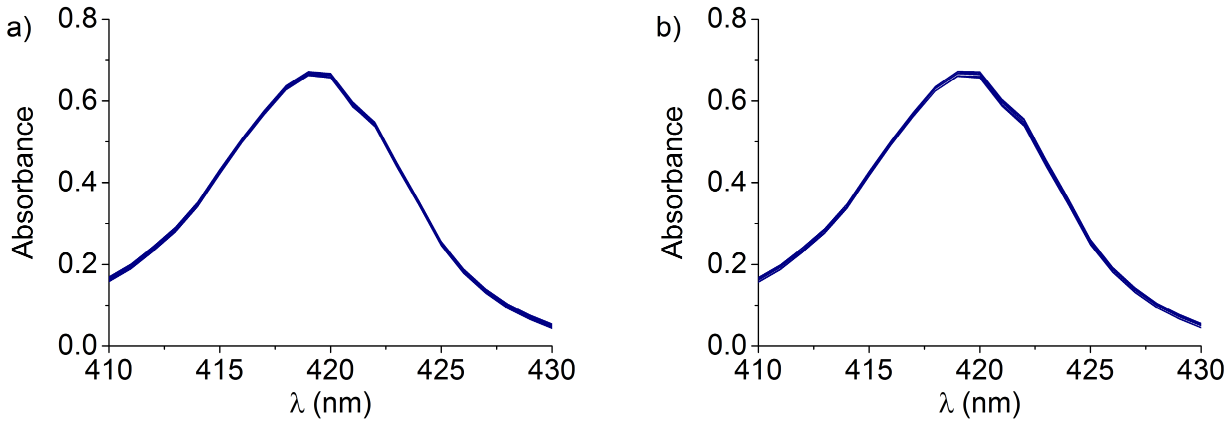


**Fig S27**. UV-visible absorbance spectra of 1.5 μM solutions of compound **16**·$PF_6$ in DMSO recorded after progressive addition of increasing concentrations of a) TBABr and b) TBAI. Final anion concentrations: 5 mM

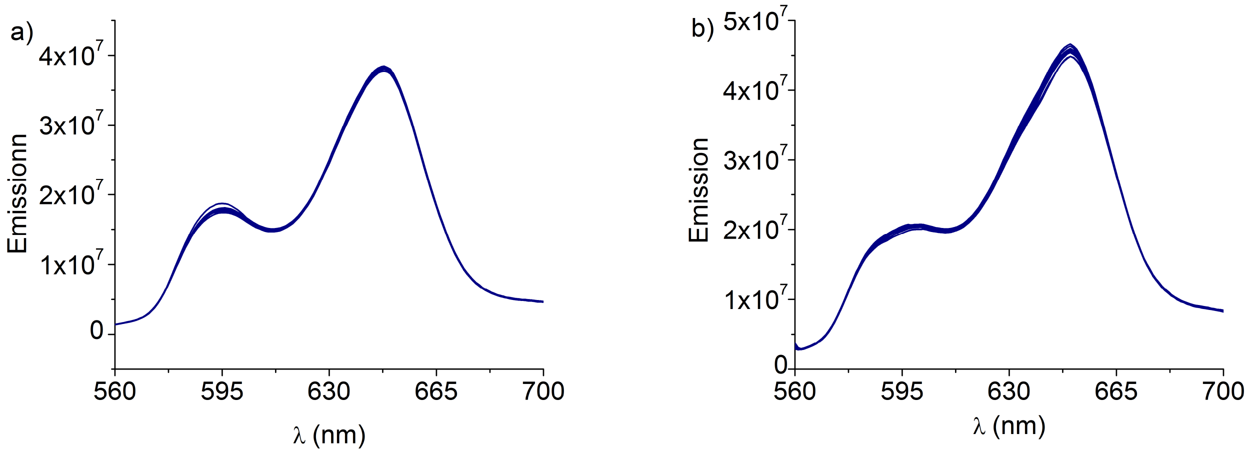


**Fig S28**. Fluorescence emission spectra of 40 μM solutions of compound **16**·$PF_6$ in DMSO recorded after progressive addition of increasing concentrations of a) TBABr and b) TBAI. Final anion concentrations: 6.1 mM

## References

[1] J. S. Manka, D. S. Lawrence, *Tetrahedron Lett.* **1989**, *30*, 6989-6992.

[2] a) Goldenbe.C, Wandestr.R, F. Binon, R. Charlier, *Chim. Ther.* **1973**, *8*, 259-270; b) A. Benarab, S. Boyé, L. Savelon, G. Guillaumet, *Tetrahedron Lett.* **1993**, *34*, 7567-7568.

[3] M. R. Sambrook, P. D. Beer, J. A. Wisner, R. L. Paul, A. R. Cowley, *J. Am. Chem. Soc.* **2004**, *126*, 15364-15365.

[4] M. J. Hynes, *J. Chem. Soc., Dalton Trans.* **1993**, 311-312.

[5] Specfit *v.* 2.02, Spectrum Software Associates Chapel Hill, NC, USA.
